# The R plasmid pRAS2 in the fish pathogen *Aeromonas salmonicida* is a global mediator of antibiotic resistance in aquaculture and can be used by pRAS3 for mobilization

**DOI:** 10.3389/fmicb.2026.1872994

**Published:** 2026-09-17

**Authors:** Surya Prasad Dahal, Hetron Mweemba Munang’andu, Asta Solberg, Henning Sørum

**Affiliations:** 1 Faculty of Biosciences and Aquaculture, Nord University, Bodø, Norway; 2 Department of Paraclinical Sciences, Faculty of Veterinary Medicine, Norwegian University of Life Sciences, Ås, Norway

**Keywords:** *Aeromonas salmonicida*, antimicrobial resistance, aquatic microbiology, conjugative plasmids, helper-dependent mobilization, horizontal gene transfer, plasmid ecology, type IV secretion system

## Abstract

Horizontal gene transfer (HGT) mediated by conjugative plasmids is a major driver of antimicrobial resistance (AMR) dissemination in aquatic environments. In *Aeromonas salmonicida*, plasmid-mediated HGT is largely driven by the pRAS plasmid family. Here we present genomic and experimental characterization of pRAS2 from *A. salmonicida* strain 1682/92 and demonstrate its role in helper-dependent mobilization of the co-resident pRAS3. Whole genome sequencing (WGS) divided the pRAS2 backbone into three modules. Module A encodes replication, maintenance and inheritance stability functions, including transposase, resolvase, partitioning and antitoxin-associated genes. Module B encodes a canonical type IV secretion system (T4SS) conjugation module containing *virB2*-*virB6*, *virB8*-*virB11* and *virD4* with *virB2* and *virB5* forming the transfer pilus. Module C encodes the resistance determinants *sul2*, *aph(6)-Id*, *neo* and Tet 31. Modules A and B were organized as in plasmids of *Vibrio alginolyticus*, *Edwardsiella piscicida* and *Piscirickettsia salmonis*, indicating that pRAS2 shares a conserved backbone with plasmids of other aquatic bacteria, including fish pathogens isolated on different continents. Module C was the most variable region: *sul2* and *aph(6)-Id* were shared with pEIB202 of *E. piscicida*, whereas the tetracycline resistance determinant remained the same, being Tet 31 in pRAS2 as well as in pEIB202, p3PS10 and p3PS11, but absent from pVb1978 (*Vibrio alginolyticus*), pVIM-20710 (*Enterobacter hormaechei*) and p4PS9. The pRAS3 plasmids of strains 1682/92 and 2131/90, used as donors in conjugation experiments with an *Escherichia coli* DH5α recipient, carried the same mobilization-associated (MOB) genes and the tetracycline resistance determinant Tet C. Transfer of pRAS3 occurred only from strain 1682/92, which carried both pRAS2 and pRAS3; no transconjugant colonies were recovered from strain 2131/90, which carried pRAS3 alone. WGS showed an identical organization of the MOB, *tetA*(C) and *tetR*(C) genes in the 1682/92 donor and its transconjugant, providing experimental evidence that pRAS2 functions as a conjugative helper plasmid mediating mobilization of pRAS3. Conjugation trials performed with these strains between 1993 and 1995 further show that pRAS2 is self-transmissible, transferring autonomously at a frequency approximately three orders of magnitude higher than that of its co-mobilization of pRAS3. This demonstrates plasmid-plasmid cooperation within the pRAS plasmid family as a mechanism supporting the spread of AMR genes in aquatic bacterial communities.

## 1 Introduction

Antimicrobial resistance (AMR) in pathogenic bacteria is one of the greatest global health threats facing humanity, husbandry- and pet animals and farmed aquatic organisms. AMR is mostly driven by misuse and overuse of antibiotics (WHO, 2022). In aquaculture, antibiotics are widely used for the treatment of bacterial diseases resulting in increase of AMR (Schar et al., 2018; Watts et al., 2017). In Norway the aquaculture industry mostly depended on the use of antibiotics to combat bacterial pathogens in the 1980s and early 1990s. Furunculosis caused by *Aeromonas salmonicida* subspecies *salmonicida* was the most dominant disease in the early 1990s (Sørum and L’Abée-Lund, 2002). Furunculosis emerged as an important disease adversely affecting the Norwegian aquaculture industry from 1988 through 1995 after importing infected smolts from Scotland (Izadi and Vajargah, 2022). Initially, outbreaks were controlled by using antibiotics like oxytetracycline, sulphadiazine and trimethoprim that led to development of multidrug-resistant (MDR) *A. salmonicida* subspecies *salmonicida* strains (Sørum and L’Abée-Lund, 2002; Sørum et al., 2003). After development of antibiotic resistance against tetracycline, sulphonamides and trimethoprim, quinolones such as oxolinic acid and then flumequine were used. Point mutations in the *gyrA* gene of *Aeromonas salmonicida* subspecies *salmonicida* developed quickly (Oppegaard and Sørum, 1994) and when an effective vaccine was developed just before the mid-90’s little effect of the medical use of antibiotics against furunculosis was left. By the late 1980s, plasmids expressing oxytetracycline, sulphonamide, and trimethoprim resistance were isolated from *A. salmonicida* infected fish (Sørum et al., 2003). By the mid-1990s, the high economic losses caused by furunculosis led the Norwegian aquaculture industry to adopt a rigorous vaccination program that dramatically reduced both the incidence of furunculosis, and antibiotic use in subsequent years (Markestad and Grave, 1997). Still, *A. salmonicida* infections linked to AMR have continued to cause economic losses in the aquaculture industry in different countries around the world (Attéré et al., 2023; Figueroa et al., 2019; Godoy et al., 2010; Hänninen et al., 1995; Inglis et al., 1991; Lin et al., 2020; McIntosh et al., 2008; Rostang et al., 2025). Because much of this resistance was plasmid-borne, the pRAS family of resistance plasmids first characterized in the Norwegian outbreaks has since emerged as globally distributed vehicles for the dissemination of antimicrobial resistance in aquaculture.

Conjugative transfer of plasmids is the most efficient mode of horizontal gene transfer (HGT), which has significantly contributed to the spread of AMR among bacteria. Conjugative plasmids transfer DNA from donor to recipient bacteria using a specialized multi-protein complex called the conjugation apparatus (Grohmann et al., 2018; Partridge et al., 2018). In *A. salmonicida*, plasmid-mediated HGT is largely associated with the pRAS family of plasmids (Sørum et al., 2003). From the Norwegian isolates collected during these outbreaks, Sørum and coworkers identified and characterized several pRAS plasmids encoding trimethoprim, sulphonamide and tetracycline resistance (L’Abée-Lund and Sørum, 2000; Sørum et al., 2003). In their studies (L’Abée-Lund and Sørum, 2000; Sørum et al., 2003), they found the pRAS1 that contained the Tet A determinant causing tetracycline resistance, the *dfr16* gene causing trimethoprim resistance and *sul1* causing sulphonamide resistance and the pRAS2 plasmid that can cause streptomycin (*strB/aph(6)-Id*), sulphonamide *(sul2*), and tetracycline (*tetA*(31)) resistance. They also isolated the pRAS3.1 and pRAS3.2 plasmids that carried the tetracycline resistance determinant Tet C together with the mobilization-associated (MOB) genes. Altogether, Sørum and coworkers (L’Abée-Lund and Sørum, 2002) identified four types of pRAS plasmids from the Norwegian *A. salmonicida* isolates. Based on conjugation experiments carried out in their studies, Sørum and coworkers observed that the three largest plasmids pRAS1, pRAS2, and pRAS4 that varied from approximately 45–50 kb were all self-transmissible, whereas pRAS3 differed from the other plasmids by being small and non-conjugative. Their studies also showed that the conjugative pRAS plasmids were transferable not only between *A. salmonicida* strains, but also to other species such as *Escherichia coli*, indicating that these plasmids cross species boundaries (Kruse and Sørum, 1994). The occurrence of plasmids closely related to pRAS3 in *Chlamydia suis* (Schneider et al., 2019), together with reports of closely related resistance plasmids in aeromonads from aquaculture and clinical sources (Cabello et al., 2013; Rhodes et al., 2000) further raises the possibility that plasmids of aquatic bacteria act as reservoirs of AMR determinants accessible to bacteria of terrestrial hosts.

L’Abée-Lund and Sørum found the coexistence of the non-conjugative pRAS3 with either pRAS1 or pRAS2 conjugative plasmids in some *A. salmonicida* isolates (L’Abée-Lund and Sørum, 2002). They noted that pRAS3 was only transferrable when it coexisted with pRAS1 or pRAS2, but it was not transferable when it was residing without pRAS1 or 2 (L’Abée-Lund and Sørum, 2002). They further showed that the pRAS2 of *A. salmonicida* subspecies *salmonicida* strain 1682/92 contained the Tn*5393*-like transposon and the *strA-strB* genes mostly found in small non-conjugative plasmids (Sundin and Bender, 1995). It has been shown that conjugative plasmids are self-transmissible from one bacterial cell to another, while mobilizable plasmids are only transferrable using the help of a conjugative plasmid co-residing in the same bacterial cell (O’Brien et al., 2015).

Despite these observations, the underlying mechanisms by which non-conjugative plasmids like pRAS3 are transferred have not been elucidated among the pRAS plasmids. The composition and organization of the complete structure of the genes forming the pRAS2 conjugative system have not been determined. It is unknown whether pRAS2 encodes a complete T4SS conjugation system as seen in other plasmids.

This study had three aims (i) to resolve the modular genomic structure of pRAS2 and identify its complete T4SS conjugation gene set; (ii) to verify experimentally, using controlled conjugation assays, that pRAS2 is required as a helper plasmid for the mobilization of pRAS3; and (iii) to perform cross-species comparative genomics on pRAS2-like plasmids in order to establish the distribution of this conserved conjugative backbone in aquaculture environments.

## 2 Materials and methods

### 2.1 Bacterial isolates and culture

The *A. salmonicida* isolates used in this study, strains 1682/92, 2298/92 (historical wet-lab studies) and 2131/90, were resistant to tetracycline, compound sulphonamides, and trimethoprim. *A. salmonicida* subsp. *salmonicida* strain 1682/92 is an isolate that caused typical furunculosis in salmon in 1992 in Sogn and Fjordane County on the West Coast of Norway (L’Abée-Lund and Sørum, 2000), whereas strain 2131/90 (Sørum et al., 2000) was isolated from diseased Atlantic salmon (*Salmo salar* L.) with atypical furunculosis in 1990 in Hordaland County also on the West Coast of Norway (L’Abée-Lund and Sørum, 2000). The difference between the two strains from fish with typical (1682/92) and atypical (2131/90) furunculosis, respectively, is that strain 1682/92 encoded two R plasmids, namely the pRAS2 and pRAS3.1 (L’Abée-Lund and Sørum, 2002) while the atypical *A. salmonicida* strain 2131/90 is encoding pRAS3.2 as the only R plasmid. Both strains were used as donors in the conjugation experiments. On the other hand, *E. coli* (DH5α) procured from ThermoFischer Scientific Life technologies was used as the recipient in the conjugation experiments. Both *A. salmonicida* strain 1682/92 and 2131/90 were grown on blood agar at 15°C for 72 h while the culture of *E. coli* DH5α was prepared at 37°C on blood agar for 24 h in an aerobic incubator.

### 2.2 Antimicrobial susceptibility testing (AST)

Both donor *A. salmonicida* strains 1682/92 and 2131/90, together with the recipient *E. coli* DH5α, were tested for antimicrobial susceptibility using a standard agar disc diffusion test as described by Kirby-Bauer (Hudzicki, 2009) and the National Committee for Clinical Laboratory Standards (Kiehlbauch et al., 2000). Mueller-Hinton (MH) agar plates were used as the growth medium, and antibiotic susceptibility discs (Oxoid, United Kingdom) were applied to the surface for testing susceptibility to antimicrobial agents. *A. salmonicida* donor strains were incubated at 15°C for 72 h whereas the recipient *E. coli* strain DH5α was grown at 37°C for 24 h under aerobic conditions. The antibiotic discs used contained tetracycline (TE, 30 μg), trimethoprim (W, 5 μg), streptomycin (S10, 10 μg), and compound sulphonamides (S3, 300 μg) (Oxoid, United Kingdom). The compound sulphonamides discs consisted of sulphamerazine (78.6 μg), sulphathiazole (109.4 μg), and sulphadiazine (110.5 μg) per disc, as specified by the manufacturer. Streptomycin resistance was included to assess the presence of pRAS2-associated resistance determinants, as this plasmid carries a functional Tn*5393c* transposon encoding the *strB [aph(6)-Id]* genes (L’Abée-Lund and Sørum, 2000). Testing streptomycin resistance helped verify that pRAS2 remained in the donor and was not co-transferred with pRAS3 during mobilization experiments. CLSI breakpoints (Clinical and Laboratory Standards Institute, 2008) were used for the *E. coli* recipient and transconjugant. As no standardized CLSI breakpoints are available for *Aeromonas salmonicida*, donor susceptibility was interpreted with reference to published guidelines for aquatic pathogens (Smith, 2006).

### 2.3 Minimum inhibitory concentration (MIC) determination

The minimum inhibitory concentrations (MICs) for tetracycline, trimethoprim, and sulphamethoxazole were determined using the microdilution method in Sensititre custom plates (Thermo Fisher Scientific, Waltham, MA, United States) for both *A. salmonicida* donor strains 1682/92 and 2131/90, the recipient *E. coli* DH5α, and the transconjugant 1682/92 DH5α. The results were analyzed by visual assessment of bacterial growth in wells containing specific antibiotic concentrations. After the bacterial inoculation, the final test concentrations of each suspension were adjusted to 0.5 McFarland equivalent using a Sensititre nephelometer (Thermo Fisher Scientific, Waltham, MA, United States) prior to conjugation. Microtiter plates inoculated with *A. salmonicida* were incubated at 15°C for 72 h, whereas *E. coli* transconjugants were selected by incubation at 37°C for 24 h before inspection. Each MIC was determined as the lowest concentration of antimicrobial agent inhibiting visible bacterial growth. Streptomycin MIC was not determined because streptomycin was not included in the custom Sensititre panel used in this study; therefore, streptomycin resistance was assessed by disc diffusion only.

### 2.4 Conjugation experiments and plasmid transfer analysis

Donor and recipient cultures were grown under their respective conditions and mixed at a 1:1 volume ratio. Donor *A. salmonicida* strains were cultured at 14 °C, while the recipient *E. coli* DH5α was grown at 37 °C. This temperature difference allowed selective recovery of *E. coli* transconjugants following mating, as the donor strain does not grow at 37 °C. Therefore, no additional chromosomal selection markers were required.

A loop-full of donor and recipient cultures, respectively, were mixed and spotted onto non-selective blood agar plates, followed by incubation at room temperature (∼22°C) for 24 h to facilitate cell-to-cell contact and plasmid transfer. After mating, bacterial growth from the mating area was collected, resuspended in sterile saline, and adjusted to a 0.5 McFarland standard. The suspension was then spread onto Mueller-Hinton agar plates, and antibiotic discs containing tetracycline (TE, 30 μg), compound sulphonamides (S3, 300 μg), trimethoprim (W, 5 μg) and streptomycin (S10, 10 μg) (Oxoid, United Kingdom) were placed onto the plates after drying of the agar surface. Streptomycin resistance was tested but not used as a selection marker, as the experimental design focused on detecting pRAS3 mobilization. Testing streptomycin helped confirm that pRAS2 (carrying *strB/aph(6)-Id*) remained in the donor and was not co-transferred with pRAS3. Plates were incubated at 37 °C for 24 h to selectively favor the growth of *E. coli* transconjugants while limiting the growth of the *A. salmonicida* donor. Colonies recovered from within the antibiotic inhibition zones and displaying morphology consistent with DH5α were sub-cultured on fresh plates to obtain pure isolates. Acquisition of antimicrobial resistance associated with pRAS3 was used as the primary indicator of successful plasmid transfer and was confirmed by disc diffusion and MIC testing for tetracycline and sulphamethoxazole. Conjugation frequencies, expressed as transconjugants per donor CFU (TF/donor) and per recipient CFU (TF/recipient), were calculated from three independent replicates under both broth and plate mating conditions. Representative isolates from the tetracycline and compound sulphonamides zones were selected for whole genome sequencing (WGS) to determine the plasmid content. Two donor backgrounds were examined: (1) *A. salmonicida* strain 2131/90 carrying pRAS3 only, and (2) *A. salmonicida* strain 1682/92 hosting both pRAS2 and pRAS3. Whole genome sequencing of donor strains and resulting transconjugants was used to confirm plasmid transfer during conjugation.

### 2.5 Detection and transfer testing of pRAS2 and pRAS3 in the transconjugants

Donor strains carrying pRAS3 were identified by whole genome sequencing (WGS). Conjugation assays were performed using donor strains carrying either pRAS3 alone or both pRAS2 and pRAS3. After mating, bacterial growth were recovered and plated onto Mueller Hinton agar containing antibiotic discs of tetracycline (TE, 30 μg), compound sulphonamides (S3, 300 μg), trimethoprim (W, 5 μg) and streptomycin (S10, 10 μg) (Oxoid, United Kingdom). Resistant colonies were recovered from within inhibition zones, surrounding the discs. From each antibiotic zone, colonies were collected and subjected to antimicrobial susceptibility testing (AST) to verify their resistance phenotype. Representative colonies from the tetracycline and compound sulphonamide zones were selected for WGS to determine the plasmid content. Donor *A. salmonicida* strains were cultured on blood agar at 14°C, while the recipient *E. coli* DH5α was cultured on blood agar at 37°C prior to conjugation.

### 2.6 Extraction of bacterial genomic DNA

Genomic DNA (gDNA) was extracted from *A*. *salmonicida* donors (strains 2131/90 and 1682/92) prior to conjugation experiments, as well as from transconjugants derived from the 1682/92 donor (1682/92-DH5α). No transconjugant colonies were recovered from matings involving the 2131/90 strain. DNA extraction was performed using the MagAttract^®^ HMW DNA kit according to the manufacturer’s protocols (Qiagen, Germany). Briefly, each isolate was cultured to obtain a concentration of 2 × 10^9^ CFU/mL. Freshly grown bacterial cultures were centrifuged in 2 mL Eppendorf tubes. The resulting pellets were resuspended in 180 μL ATL buffer, followed by the addition of 20 μL Proteinase K. Samples were incubated at 56 °C in an Eppendorf thermomixer for 30 min. Subsequently, 4 μL of RNase was added, and samples were pulse vortexed before adding 15 μL MagAttract Suspension G and 280 μL Buffer MB. The suspensions were transferred onto a MagAttract magnetic holder and vortexed for 60 s. Magnetic beads containing gDNA were separated by placing the tubes on the magnetic rack for 60 s, allowing removal of the supernatant without disturbing the beads. The beads were washed twice using wash buffer (MW1) and phenol/ethanol (PE) buffer. Residual contaminants were removed by rinsing the beads twice with 1 mL distilled water. The purified gDNA was eluted in 100 μL elution buffer (EB). DNA purity was assessed using the NanoDrop spectrophotometer (Thermo Fisher Scientific, Waltham, MA, United States) and agarose gel electrophoresis (1 %). DNA concentration was measured using the Qubit double-stranded DNA (dsDNA) HS kit according to the manufacturer’s instructions (Life Technologies Inc., CA, United States).

### 2.7 Library preparation and whole genomic sequencing

Sequence libraries were prepared for the two donors *A. salmonicida* strains (1682/92 and 2131/90) and for the transconjugant derived from the 1682/92 donor (1682/92-DH5α) using the Nextera DNA Flex Tagmentation kit (Illumina Inc., San Diego, CA, United States) (Gaio et al., 2022). The *E. coli* DH5α recipient was not sequenced in this study; the reference assembly GCF_000982435.1 was used to confirm the absence of pRAS2- and pRAS3-related sequences from the recipient background. Libraries were quantified using the Qubit^®^ DNA HS Assay Kit with a Qubit fluorometer (Thermo Fisher Scientific, Waltham, MA, United States). Fragment size distribution was assessed using the Agilent HS DNA Kit on the Agilent 2100 Bioanalyzer (Agilent Technologies, CA, United States). Sequencing was performed on an Illumina MiSeq platform (Illumina Inc., United States) using the v3 reagent kits with a paired-end read length of 2 × 301 bp (Kaspersen et al., 2020). Mean sequencing depth was estimated as the total number of raw bases (read pairs × 2 × 301 bp) divided by the size of the corresponding assembly, giving 61× for strain 1682/92, 36× for strain 2131/90 and 140× for the transconjugant 1682/92-DH5α. Bioinformatic analysis was conducted using the Galaxy platform (version 21.05) (The Galaxy Community, 2024). Raw reads were quality-checked using FastQC (v0.11.9) (Andrews, 2010), and adapter trimming and quality filtering were performed using Trimmomatic (v0.38.1) (Bolger et al., 2014). The filtered reads were assembled to remove the adapters and low-quality reads from paired end sequences. The resulting paired-end sequence reads were *de novo* assembled into contigs using A5-miseq assembler (Coil et al., 2014) of which quality sequence contigs with 33-91 k-mers were assembled using SPAdes (v3.12.0) (Bankevich et al., 2012). Genome annotation was performed using Prokka (Seemann, 2014), and results were compared with annotations generated using the NCBI Prokaryotic Genome Annotation Pipeline (PGAP) (Tatusova et al., 2016).

### 2.8 Prediction of plasmids and antimicrobial resistance genes

Identification of AMR genes was done in Staramr version 0.7.2 and ABRicate version 1.0.1 in the Comprehensive Antimicrobial Resistance Database (CARD) software at a threshold of 80% (Alcock et al., 2020). Identification of plasmids was done using PlasmidFinder v 2.0 at a threshold of 80% (Carattoli et al., 2014). Proksee was used to generate circular maps (Grant et al., 2023).

### 2.9 Statistical analysis

Conjugation frequencies from three independent biological replicates per mating condition were log_10_-transformed prior to analysis. Broth and plate mating conditions were compared by unpaired two-tailed Student’s *t*-test on the log_10_- transformed values using R version 4.3.2. A *p*-value below 0.05 was considered statistically significant. Data are presented as mean ± SD of the untransformed frequencies.

## 3 Results

### 3.1 Antimicrobial susceptibility

Among the *A. salmonicida* donor strains tested using the disc diffusion method, strain 1682/92 was resistant to tetracycline, compound sulphonamides and streptomycin but susceptible to trimethoprim, whereas 2131/90 was resistant to tetracycline but susceptible to compound sulphonamides, trimethoprim and streptomycin (Table 1). Trimethoprim was included in the susceptibility panel to assess the potential presence of trimethoprim resistance determinants commonly associated with multidrug resistance plasmids in *A. salmonicida*, although neither donor strain exhibited phenotypic resistance. The recipient *E. coli* DH5α was susceptible to all four antibiotics tested. The transconjugant derived from strain 1682/92 (1682/92-DH5α) exhibited resistance to tetracycline but remained susceptible to compound sulphonamides, trimethoprim and streptomycin, indicating selective acquisition of tetracycline resistance without co-transfer of the pRAS2 associated determinants. No transconjugant colonies were recovered from matings involving the 2131/90 donor strain. These results are consistent with mobilization of pRAS3 from the 1682/92 donor.

**TABLE 1:** Disc diffusion antibiotics susceptibility profiles of donors, recipient and transconjugant.

Strain	Tetracycline	Compound sulphonamides	Trimethoprim	Streptomycin
*A. salmonicida* 1682/92 (donor)	R	R	S	R
*A. salmonicida* 2131/90 (donor)	R	S	S	S
*E. coli* DH5α (recipient)	S	S	S	S
1682/92-DH5α (transconjugant)	R	S	S	S

R, resistant; S, susceptible, interpreted as described in section 2.2.

### 3.2 Minimum inhibition concentration

Table 2 shows the MICs for tetracycline, sulphamethoxazole, and trimethoprim determined for both donor strains, the recipient *E. coli* DH5α, and the transconjugants. Among the donors, strain 1682/92 showed MICs of 32 μg/mL for tetracycline, 8 μg/mL for sulphamethoxazole, and ≤ 0.25 μg/mL for trimethoprim. In contrast, strain 2131/90 showed tetracycline MIC of 32 μg/mL but remained susceptible to sulphamethoxazole and trimethoprim ( ≤ 0.25 μg/mL), consistent with its disc diffusion profile. The recipient *E. coli* DH5α showed MICs at or below the lowest concentrations tested for all three antibiotics ( ≤ 0.25 μg/mL sulphamethoxazole, ≤ 0.25 μg/mL trimethoprim, and ≤ 0.25 μg/mL tetracycline). The transconjugant 1682/92-DH5α exhibited tetracycline MIC (32 μg/mL) identical to the donor strain 1682/92 but remained susceptible to sulphamethoxazole and trimethoprim ( ≤ 0.25 μg/mL), indicating selective transfer of tetracycline resistance determinants.

**TABLE 2:** Minimum inhibitory concentrations for the donors, recipient, and transconjugant strains.

Strain	Tetracycline (μg/mL)	Sulphamethoxazole (μg/mL)	Trimethoprim (μg/mL)
*A. salmonicida* 1682/92	32	8	≤ 0.25
*A. salmonicida* 2131/90	32	≤ 0.25	≤0.25
*E. coli* DH5α	≤ 0.25	≤0.25	≤ 0.25
1682/92-DH5α	32	≤ 0.25	≤0.25

MIC values represent the lowest concentration inhibiting visible growth. Values reported as ≤ indicate inhibition at the lowest concentration tested. Streptomycin was not included in the Sensititre panel and was assessed by disc diffusion only.

### 3.3 Whole genome sequencing of the donor, recipient and transconjugant bacteria

Table 3 summarizes the results of WGS for the two donors and the transconjugant, together with the reference assembly of the *E. coli* DH5α recipient. The assembled genome sizes of the donor strains 1682/92 and 2131/90 were estimated at 4.69 Mb and 4.32 Mb, respectively. Both donor strains exhibited higher GC content than the *E. coli* recipient, and strain 1682/92 showed a higher number of predicted proteins and genes than strain 2131/90 (Table 3). WGS analysis revealed that strain 1682/92 harbored both pRAS2 and pRAS3 (Figures 1, 2). In contrast, strain 2131/90 did not carry pRAS2 but contained a plasmid with high sequence similarity to pRAS3 (Figure 3), together with the species-specific plasmids. The *E. coli* DH5α reference assembly (GCF_000982435.1, 4.51 Mb) contains no sequences corresponding to pRAS2 or pRAS3, confirming that these plasmids were absent from the recipient background. WGS of the transconjugant 1682/92-DH5α identified a contig corresponding to pRAS3, with gene content and organization matching the pRAS3 of the 1682/92 donor (Figure 4), whereas no contigs corresponding to pRAS2 were detected. This indicates that pRAS3 was mobilized into the recipient while pRAS2 was not transferred. No transconjugants were recovered from matings involving strain 2131/90, and therefore no corresponding transconjugant genome was included in the analysis.

**TABLE 3:** Genome data of the donors, recipients and transconjugants.

Strain	Size Mb	GC content	Scaffold	Proteins	Genes	R-plasmid	NCBI number
*A. salmonicida* 1682/92	4.69	58.54	92	4,339	4,489	pRAS3+pRAS2	SAMN54892334
*A. salmonicida* 2131/90	4.32	59.12	185	4,085	4,173	pRAS3	SAMN54892336
*E. coli* DH5α	4.51	50.74	89	4,201	4,281	None	GCF_000982435.1
1682/92-DH5α	4.54	50.77	63	4,220	4,301	pRAS3	SAMN54892337

**FIGURE 1:**
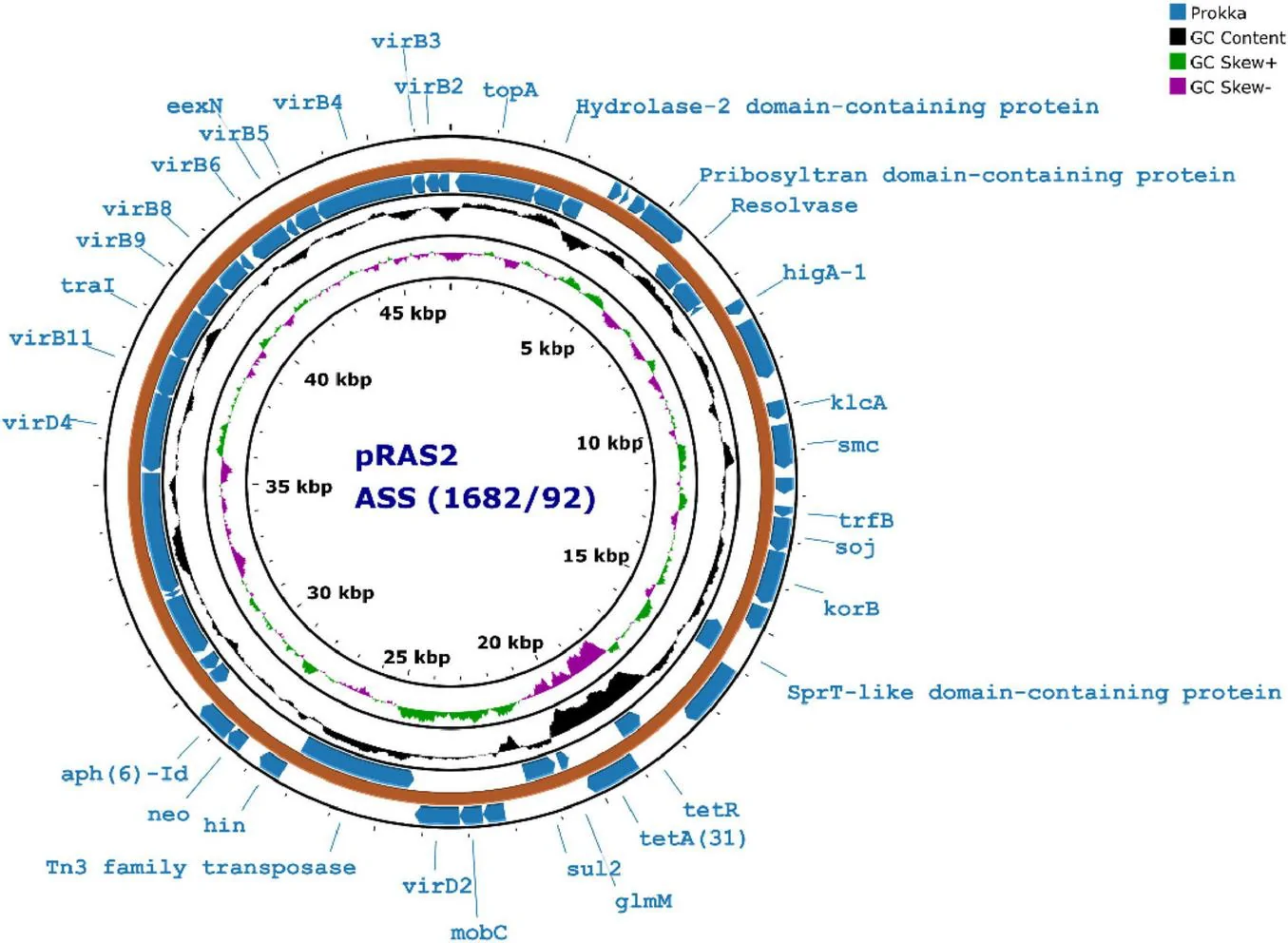
Circular genome map of the pRAS2 plasmid (pRAS2-1682/92) from *A. salmonicida* subsp. *salmonicida* (strain 1682/92), showing antimicrobial resistance genes, replication and maintenance genes, and conjugation/transfer genes. Inner rings represent GC content and GC skew.

**FIGURE 2:**
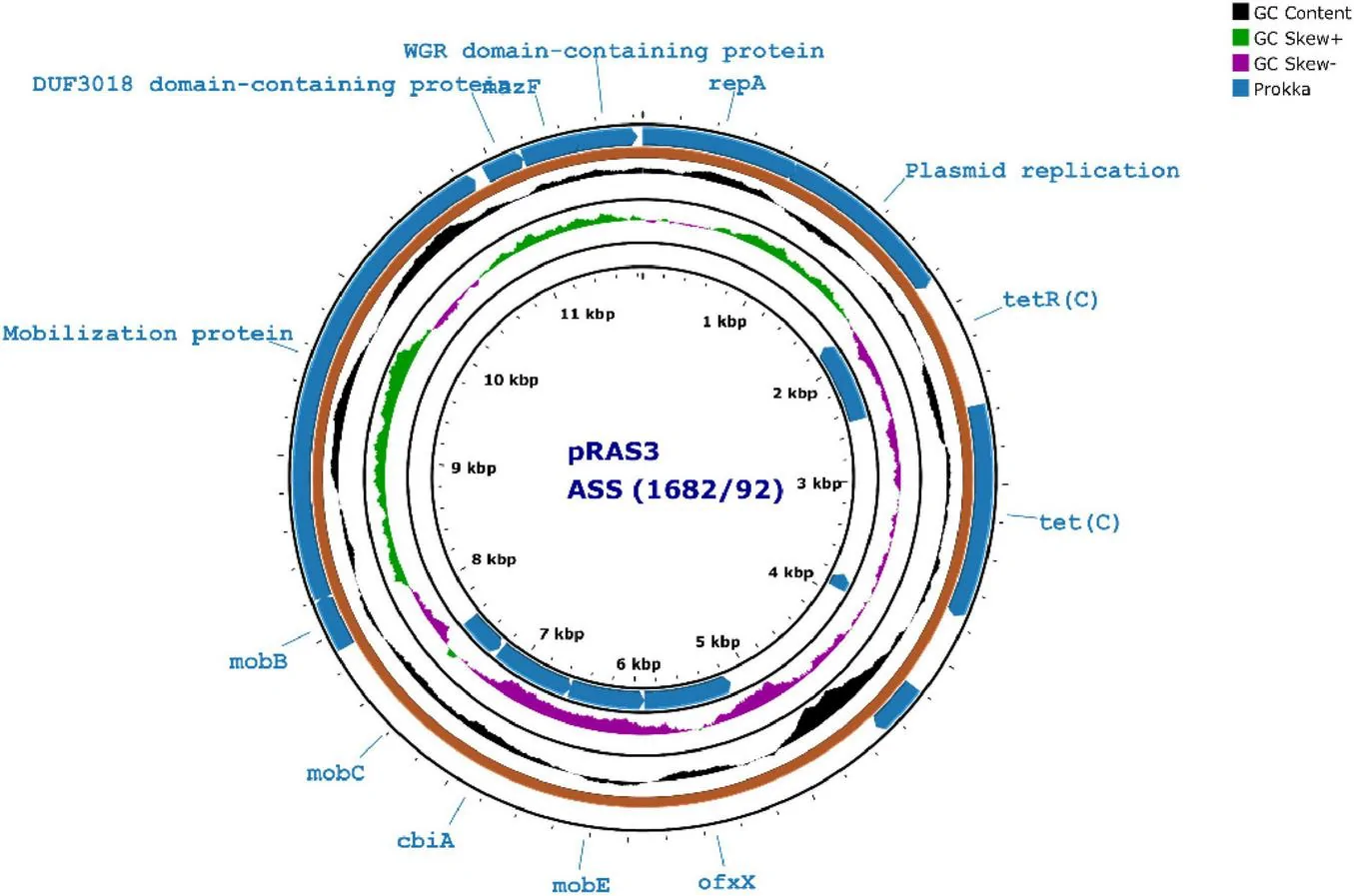
Circular map of the pRAS3 plasmid (pRAS3-1682/92) from *Aeromonas salmonicida* subsp. *salmonicida* strain 1682/92.

**FIGURE 3:**
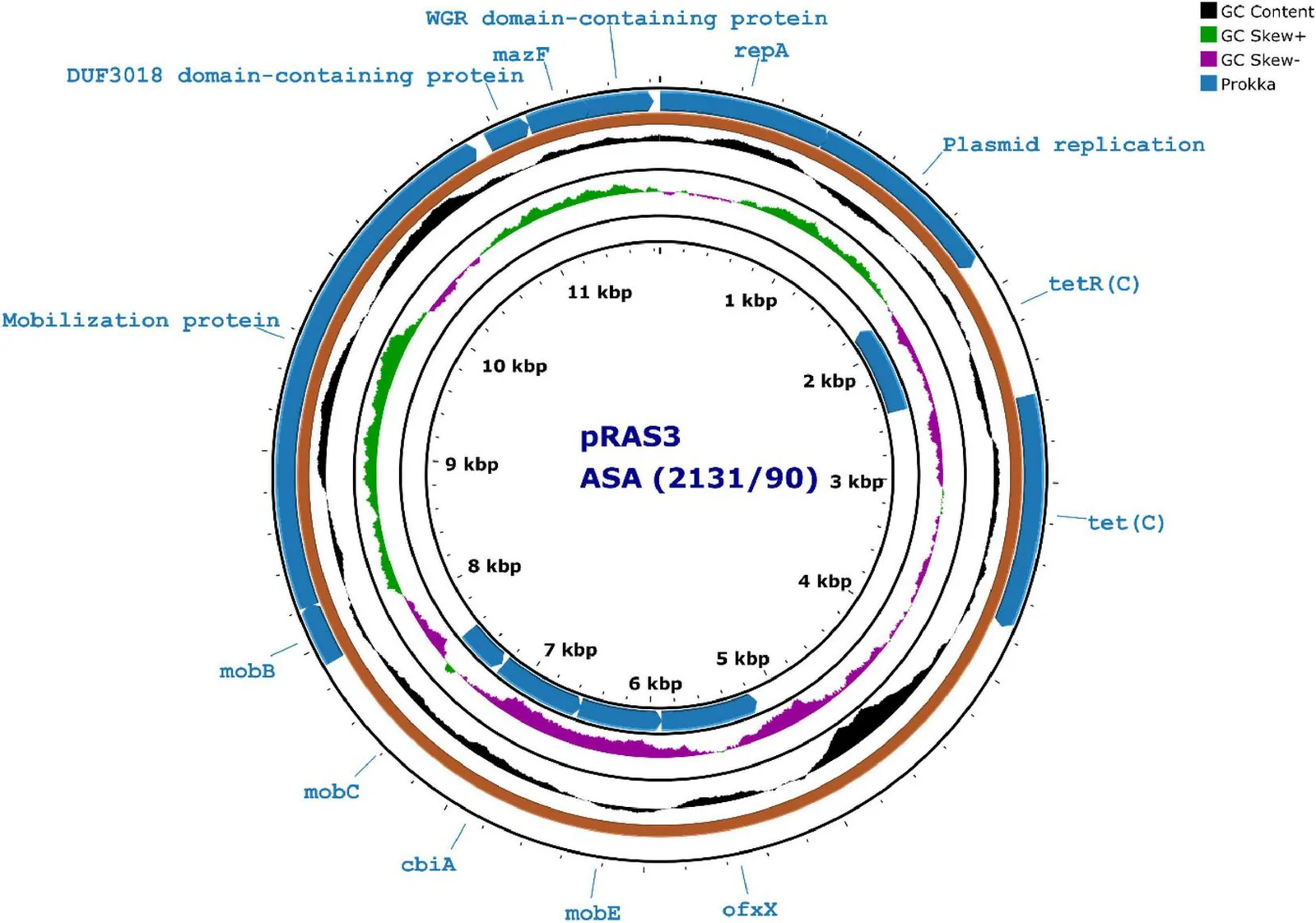
Circular map of the pRAS3-2131/90 plasmid from *Aeromonas salmonicida* strain 2131/90.

**FIGURE 4:**
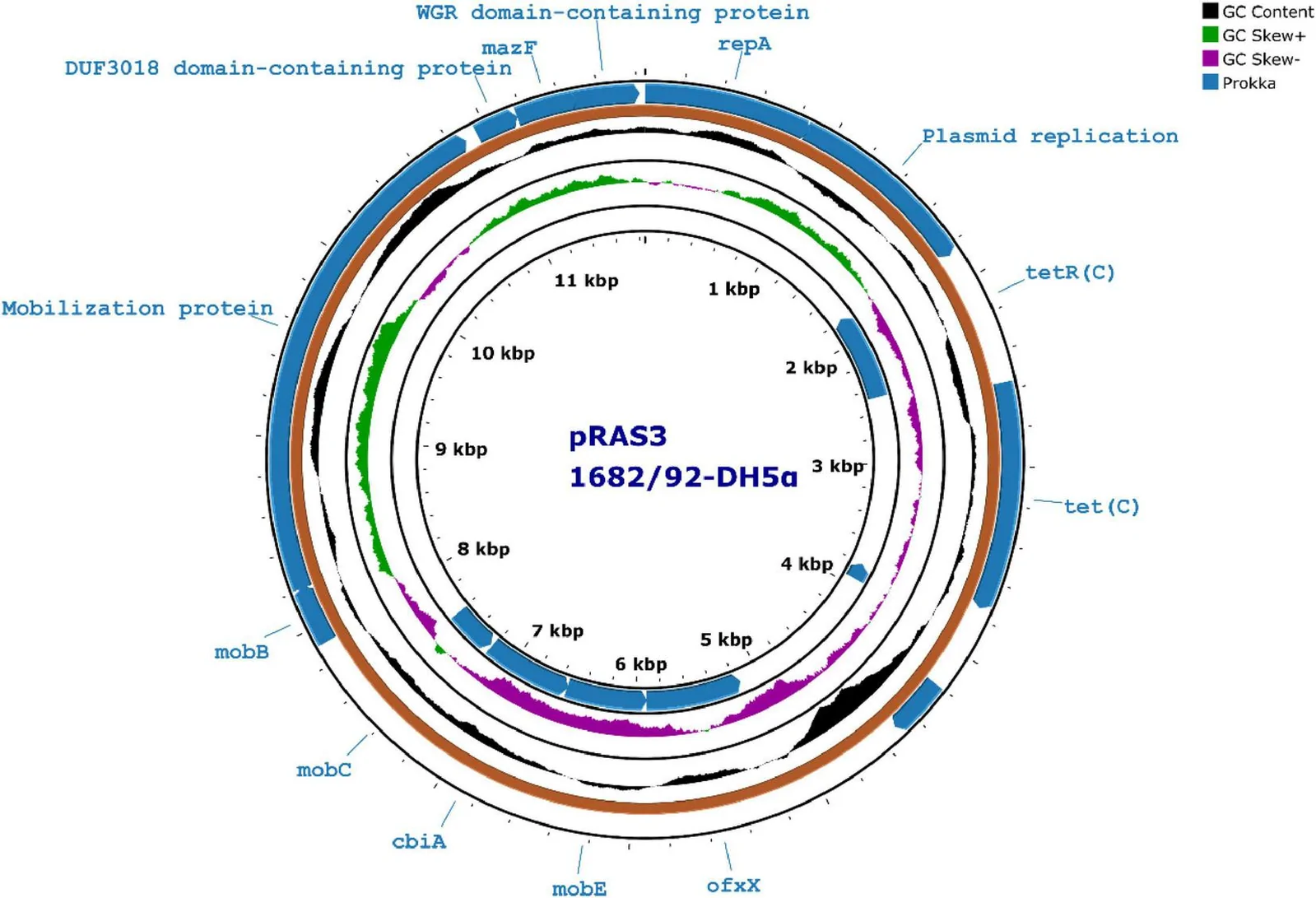
Circular map of the pRAS3 plasmid recovered from the transconjugant 1682/92-DH5α, obtained by the mating of the donor *Aeromonas salmonicida* strain 1682/92 with the *E. coli* DH5α recipient. The map shows the mobilization (*mob*) genes, the TetA(C) efflux pump and its TetR(C) repressor, and the replication region.

### 3.4 Transfer frequency of pRAS3 mobilization

To quantify the efficiency of pRAS3 mobilization, conjugation frequencies were determined under both broth and plate mating conditions using standardized donor-recipient mixtures (Figure 5). Transfer frequencies were 6.65 × 10^−7^ ± 1.98 × 10^−7^ for broth mating and 1.08 × 10^−6^ ± 7.79 × 10^−7^ for plate mating, while TF/recipient was 5.73 × 10^−9^ ± 2.26 × 10^−9^ and 7.49 × 10^−9^ ± 1.56 × 10^−9^ respectively (*n* = 3 per condition). Neither TF/donor (*p =* 0.84) nor TF/recipient (*p* = 0.30) differed significantly between the two mating conditions. These results show that pRAS3 mobilization occurs at low but reproducible frequencies in the presence of the helper plasmid pRAS2, with donor-normalized frequencies approximately 100-fold higher than recipient-normalized frequencies.

**FIGURE 5:**
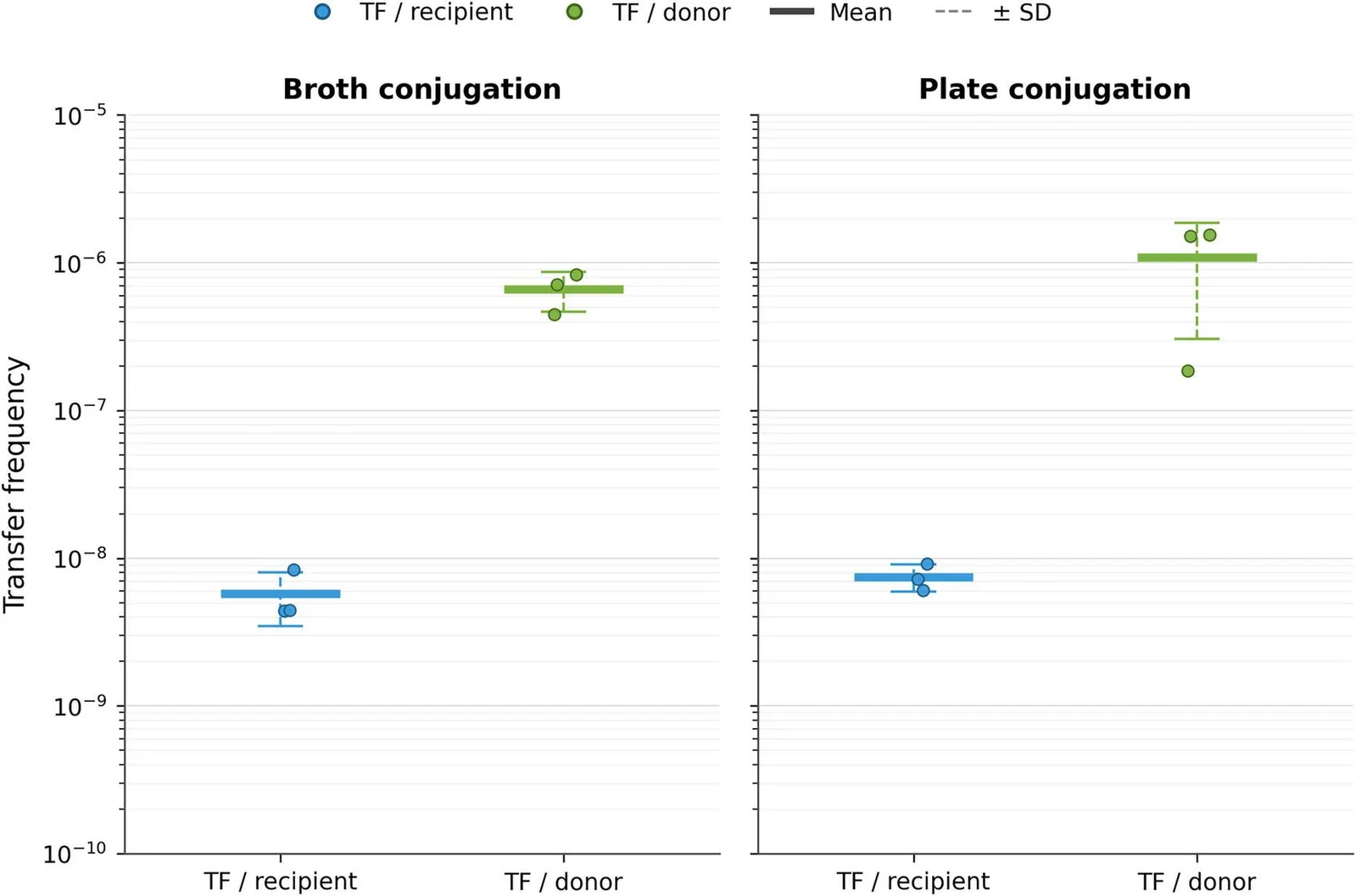
Transfer frequency of pRAS3 mobilization from *Aeromonas salmonicida* 1682/92 to *E. coli* DH5α under broth and plate mating conditions. Transfer frequencies are expressed as transconjugants per recipient (TF/recipient) and per donor (TF/donor). Circles represent individual biological replicates (*n* = 3), solid lines indicate the mean and dashed lines represent ± SD. Y-axis is shown on a log_10_ scale. Differences between broth and plate mating conditions were not significant (*p =* 0.84 for TF/donor and *p* = 0.30 for TF/recipient; unpaired two-tailed *t*-test on log_10_- transformed values.

### 3.5 Characterization of the pRAS2 plasmid in typical *Aeromonas salmonicida* strain (1682/92)

Analysis of the composition and organization of genes that are forming the pRAS2 backbone in *A. salmonicida* showed that this plasmid can be divided into three modules (Figure 6). Module A comprises genes involved in plasmid replication, maintenance, and stable inheritance. These include *tnpA* (encoding a transposase), *sprT* (small protein T), *res* (resolvase), *korB* (kil-override protein B), *soj* (chromosome partitioning protein), *trfB* (transcriptional regulator), *smc* (structural maintenance of chromosome protein), *klcA* (kilA protein), *repA* (replication initiator protein), *higA* (antitoxin protein), *traX* (transfer-associated protein), *bin3* (bridging integrator protein) and *prt* (phosphoribosyl transferase) (Figure 6).

**FIGURE 6:**
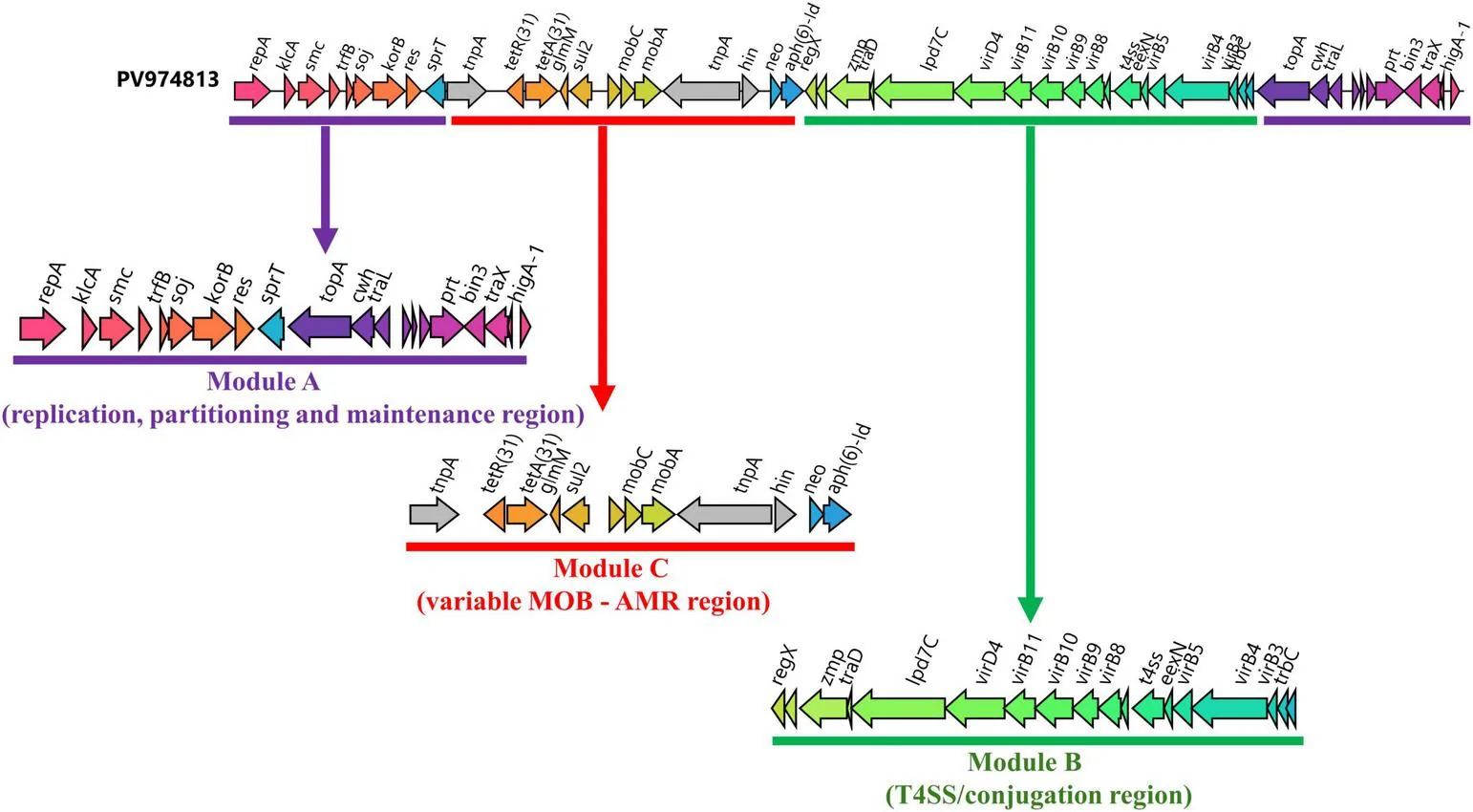
Sequence layout of the *Aeromonas salmonicida* pRAS2-1682/92 plasmid (Accession no. PV974813), showing that its genome can be divided into three modules. Module A encodes genes linked to plasmid replication, partitioning, maintenance and stable inheritance. Module B encodes *topA*, the *virB* genes and other genes associated with the T4SS mating pilus formation. Module C encodes *tnpA* and the AMR genes.

Module B comprises the conjugation-associated type IV secretion systems (T4SS), including the genes *virB2*, *virB3*, *virB4*, *virB5*, *virB6*, *virB8*, *virB9*, *virB10*, *virB11*, and *virD4* (Figure 6). Figure 7 represents a schematic representation of the predicted pRAS2 conjugation nanomachinery based on *virB* and *virD4* gene products identified in this module. In addition, Module B contains genes associated with plasmid transfer and DNA processing, including *topA, cwh, traI, traD, lpd7C*, and *eexN*.

**FIGURE 7:**
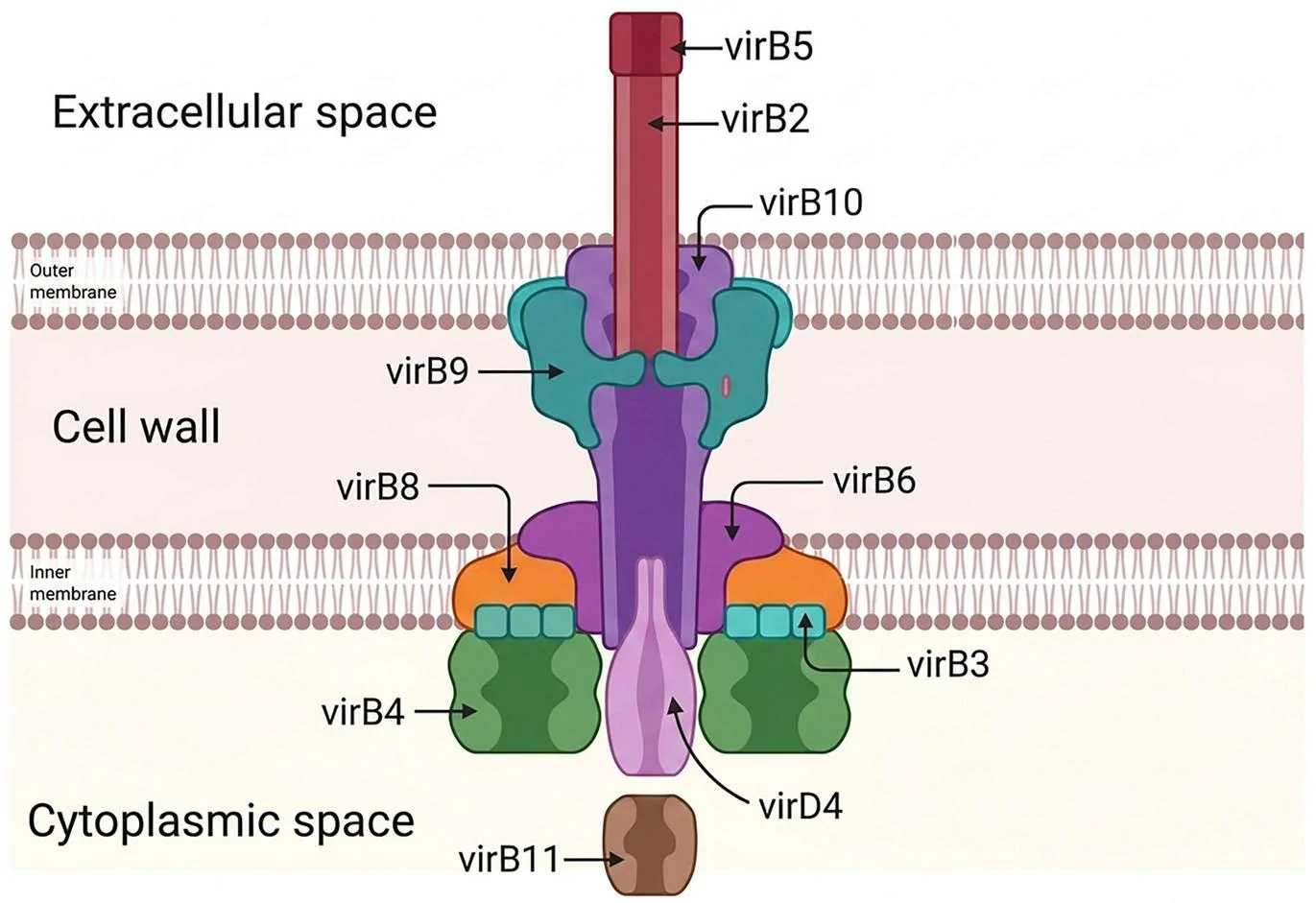
Schematic view of the hypothetical T4SS nanomachinery encoded by the pRAS2 plasmid, showing the different virB genes involved in its formation.

Module C comprises of the antimicrobial resistance region and associated mobile genetic elements. This module includes *zmp* (zinc metalloprotein), *aph(6)-Id* (*strB*) encoding streptomycin resistance, as well as *neo* (aminoglycoside resistance), *hin* (site-specific recombinase), *tnpA* (transposase), *regX* (transcriptional regulator) and the mobilization genes *mobA* and *mobC*, together with several hypothetical proteins (Figure 6). In addition, this region contains *sul2* encoding dihydropteroate synthase, *glmM* encoding phosphoglucosamine mutase, and the tetracycline resistance gene *tetA*(31) with its regulator *tetR*(31). This region corresponds to a previously described Tn*5393*-like transposon carrying streptomycin resistance genes *strB/aph(6)-Id* in pRAS2 (L’Abée-Lund and Sørum, 2000). Overall, Module C represents the variable antimicrobial resistance region of the plasmid.

### 3.6 Comparison of pRAS2 of *Aeromonas salmonicida* 1682/92 with plasmids of other bacteria

In Table 4 the plasmid sequences retrieved from Genebank that were selected for comparative analysis with pRAS2 from *Aeromonas salmonicida strain* 1682/92 are listed. These plasmids originate from diverse aquatic bacterial species, including *Piscirickettsia salmonis*, *Edwardsiella piscicida*, *Vibrio alginolyticus*, and *Enterobacter hormaechei*, and represent isolates from different geographical regions and time periods.

**TABLE 4:** Plasmid sequences from GenBank used in comparison with pRAS2 from *Aeromonas salmonicida* strain 1682/92.

Plasmid	Strain	Accession	Species	Location/Year
*pRAS2*	1682/92	PV974813.1	*Aeromonas salmonicida* subsp. *salmonicida*	Norway/1992
*p3PS10*	AY3800B	CP013819.1	*Piscirickettsia salmonis* strain	Chile/2013
*p3PS11*	AY3864B	CP013814.1	*Piscirickettsia salmonis* strain	Chile/2013
*p4PS8*	AY6297B	CP013795.1	*Piscirickettsia salmonis* strain	Chile/2015
*p4PS9*	AY6532B	CP013800.1	*Piscirickettsia salmonis* strain	Chile/2015
*pBY3*	BR19001YR	CP060033.1	*Aeromonas salmonicida* subsp. masoucida	South Korea/2018
*pEIB202*	EIB202	CP001136.1	*Edwardsiella piscicida* strain	China/2006
*pVIM-20710*	20,710	CP030081.1	*Enterobacter hormaechei* strain	China/2011
*pVb1978*	Vb1978	MG456577.1	*Vibrio alginolyticus* strain	China/1978

Comparative analysis of pRAS2-1682/92 with plasmids from other bacterial species revealed both conserved backbone regions and variable accessory elements. The closest related plasmid identified was pBY3 from *Aeromonas salmonicida* subsp. *masoucida*, which showed 99.99% nucleotide identity over 82% query coverage, indicating a highly conserved backbone. In contrast, other plasmids showed high sequence identity but low query coverage, reflecting similarity restricted to specific regions rather than the entire plasmid. For example, sequences corresponding to the Tn*5393*-like transposon and resistance-associated genes showed 100% identity but covered only 7–16% of the pRAS2 sequence. These results indicate that while the core conjugative and replication-associated regions of pRAS2 are conserved among closely related plasmids, the antimicrobial resistance regions and associated mobile genetic exhibit a modular distribution across different bacterial species. This supports the concept that pRAS2-like plasmids share a conserved backbone structure, whereas accessory regions, particularly those associated with antimicrobial resistance, are more variable and likely subject to horizontal gene transfer.

Comparative analysis of Module B (T4SS-associated genes) and Module C (AMR region) across plasmids from different bacterial species is summarized in Tables 5, 6, with gene organization illustrated in Figure 8. The organization of T4SS-associated genes in Module B of pRAS2-1682/92 is highly conserved among plasmids from *Edwardsiella piscicida* (pEIB202), *Piscirickettsia salmonis* (p3PS11 and p3PS10), *Vibrio alginolyticus* (pVb1978), and *Enterobacter hormaechei* (pVIM-20710). Sequence comparison showed high nucleotide identity in these regions, although often limited to partial query coverage, indicating conservation of specific functional modules rather than entire plasmids.

**TABLE 5:** Comparison of T4SS genes of *Aeromonas salmonicida* strain 1682/92 pRAS2 with other plasmids.

Gene	1682/92	AY3800B	AY3864B	AY6297B	BR19001YR	EIB202	20710	Vb1978
	(pRAS2)	(p3PS10)	(p3PS11)	(p4PS8)	(pBY3)	(pEIB202)	(pVIM-20710)	(pVb1978)
*traI*	**+**	**+**	**+**	**+**	**-**	**+**	**+**	**+**
*cwh*	**+**	**+**	**+**	**+**	**+**	**+**	**+**	**+**
*topA*	**+**	**+**	**+**	**+**	**+**	**+**	**+**	**+**
*virB2*	**+**	**-**	**-**	**-**	**+**	**+**	**+**	**+**
*virB3*	**+**	**+**	**+**	**+**	**+**	**+**	**+**	**+**
*virB4*	**+**	**+**	**+**	**+**	**+**	**+**	**+**	**+**
*virB5*	**+**	**+**	**+**	**+**	**+**	**+**	**+**	**+**
*virB6*	**+**	**+**	**+**	**+**	**+**	**+**	**+**	**+**
*virB8*	**+**	**+**	**+**	**+**	**+**	**+**	**+**	**+**
*virB9*	**+**	**+**	**+**	**+**	**+**	**+**	**+**	**+**
*virB10*	**+**	**+**	**+**	**+**	**+**	**+**	**+**	**+**
*virB11*	**+**	**+**	**+**	**+**	**+**	**+**	**+**	**+**
*virD4*	**+**	**+**	**+**	**+**	**+**	**+**	**+**	**+**
*lpd7C*	**+**	**+**	**+**	**+**	**+**	**+**	+	**+**
*traD*	**+**	**+**	**+**	**+**	**+**	**+**	-	**+**

Accession numbers for all plasmids are given in Table 4. + present; - absent.

**TABLE 6:** Comparison of module C genes of *A. salmonicida* 1682/92 pRAS2 with other plasmids.

Gene	1682/92 (pRAS2)	AY3800B (p3PS10)	AY3864B (p3PS11)	AY6297B (p4PS8)	BR19001YR (pBY3)	EIB202 (pEIB202)	20710 (pVIM-20710)	Vb1978 (pVb1978)
*sul2*	+	+	+	-	+	+	-	+
*aph(6)-Id*	+	-	-	-	-	+	-	-
*neo*	+	-	-	-	-	-	-	-
*tetA*(31)	+	+	+	-	+	+	-	-
*tetR*(31)	+	+	+	-	+	+	-	-
*cat/cat3*	-	-	-	-	-	+	-	+
*cmlA*	-	-	-	+	-	-	-	-
*aacA4*	-	-	-	-	-	-	-	+
*ant1*	-	+	+	+	-	-	-	+
*emrE*	-	-	-	-	-	-	-	+
*mobA*	+	+	+	-	+	+	-	-
*mobC*	+	-	-	-	+	-	-	-
*regX*	+	-	-	-	+	-	-	-
*zmp*	+	-	-	-	+	-	-	-
*glmM*	+	-	-	-	+	-	-	-
*hin*	+	-	-	-	+	-	-	-
*tnpA*	+	-	-	-	+	-	-	-
*ISKpn13*	-	+	+	+	-	-	-	-
*ISPrst3*	-	-	-	-	-	+	-	-

**FIGURE 8:**
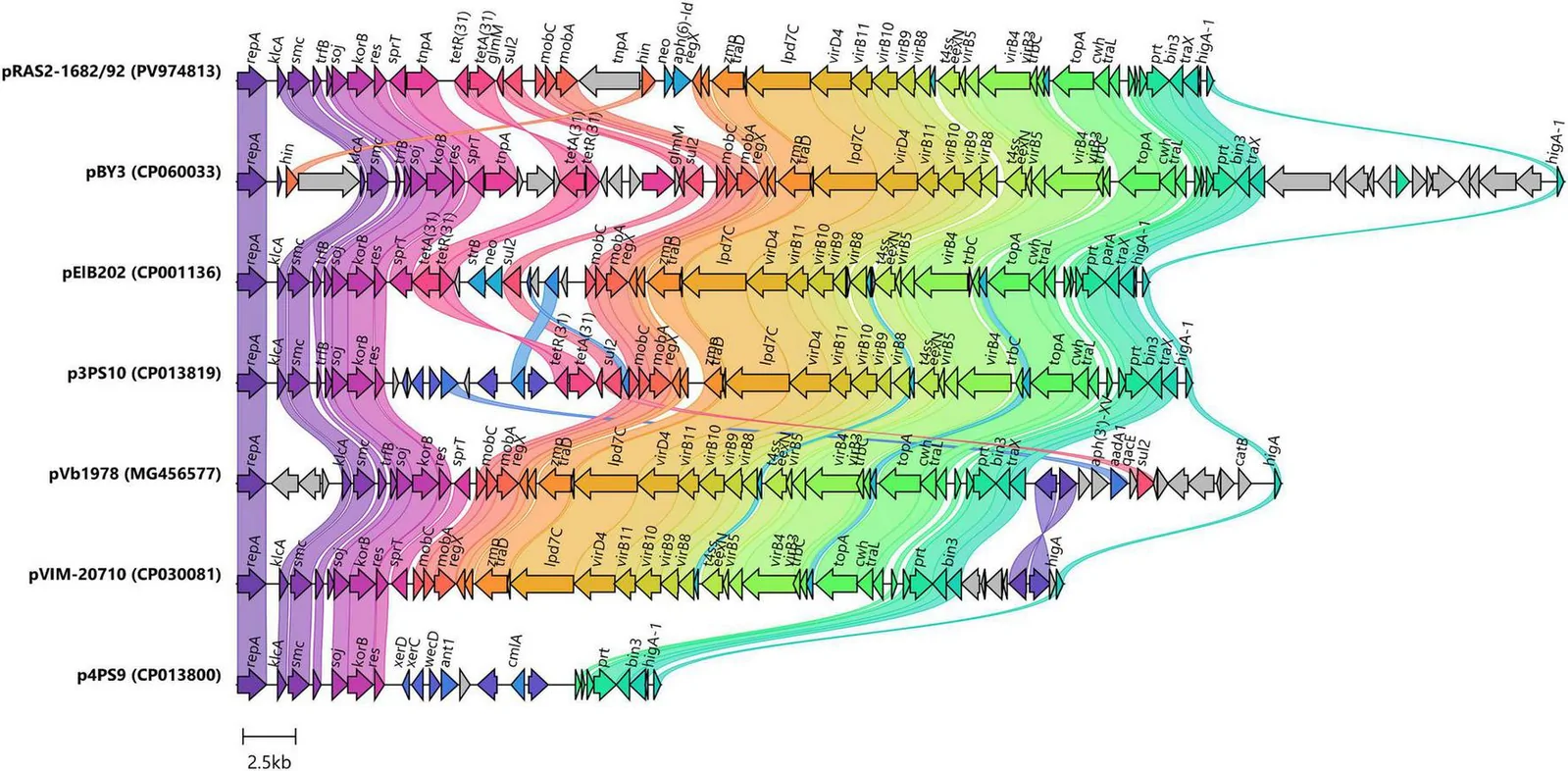
Comparison of the Norwegian *A. salmonicida* strain 1682/92 pRAS2 (accession PV974813) with, from top to bottom, pBY3 of the South Korean *Aeromonas salmonicida* subsp. *masoucida* strain BR19001YR, pEIB202 of the Chinese *Edwardsiella piscicida* strain EIB202, p3PS10 of the Chilean *Piscirickettsia salmonis* strain AY3800B, pVb1978 of the Chinese *Vibrio alginolyticus* strain Vb1978, pVIM-20710 of the Chinese *Enterobacter hormaechei* strain 20710, and p4PS9 of the Chilean Piscirickettsia salmonis strain AY6532B. Accession numbers for all plasmids are given in Table 4.

As shown in Table 5, pRAS2 (PV974813), pEIB202, and pVb1978 contain a complete set of T4SS-associated genes, including *virB2-virB6* and *virB8-virB11* together with *virD4*; *virB1* but *virB7* were not detected in any of the plasmids compared. In contrast, *virB2* is absent in p3PS10, p3PS11, and p4PS8. Additional genes present in Module B of pRAS2, including *topA*, *cwh*, *traI*, *traD*, *lpd7C*, and *eexN*, are largely conserved among the compared plasmids, although some variation is observed. For example, pBY3 lacks *traI* while pVIM-20710 lacks *traD.*

Overall, these observations indicate that the composition and organization of T4SS associated genes are conserved across plasmids from diverse aquatic bacterial species, supporting the presence of a shared conjugative backbone structure.

Module C was the most diverse of the three modules identified among plasmids from different bacterial species (Figure 8 and Table 6), encoding a range of AMR genes and transposases. In pRAS2-1682/92 (accession PV974813) of *A. salmonicida*, it contained *zmp*, *sul2*, *aph(6)-Id*, *glmM*, *hin, tnpA, regX, mobA, mobC, tetA*(31), and *tetR*(31); pBY3 (BR19001YR) carried the same set of module C genes with the exception of *neo* and *aph(6)-Id*; similarly pEIB202 (EIB202), included *tetA*(31) and its associated regulatory gene *tetR*(31), together with *aph(6*)*-Id*, *sul2*, *mobA*, *cat3*, and IS*Prst3* transposase. In p3PS11 (AY3864B) and p3PS10 (AY3800B) of *P. salmonis*, the module encoded *mobA*, *sul2*, *tetA*(31), *tetR*(31), *ant1*, and two IS*Kpn13* transposases, while in pVb1978 (Vb1978) of *V. alginolyticus*, it included *aacA4*, *ant1*, *sul2*, *cat*, and an *emrE* multi-drug transporter. In p4PS8 (AY6297B) of *P. salmonis*, it included *cmlA*, *ant1* and two IS*Kpn13* transposases. Overall, the same tetracycline resistance genes were identified in several plasmids, including *tetA*(31)/*tetR*(31) in pRAS2-1682/92, in pBY3 (BR19001YR), pEIB202 (EIB202), p3PS11 (AY3864B) and p3PS10 (AY3800B), but were absent in pVb1978 (Vb1978), p4PS8 (AY6297B), plasmids (Table 6). These observations indicate that module C encodes diverse AMR genes, with tetracycline resistance determinants conserved among a subset of plasmids.

In addition, module C shows that genetic features differ among plasmids from different bacterial species; for example, the Norwegian *A. salmonicida* strain 1682/92 contained *tnpA* and *hin*, whereas the Chilean *P. salmonis* plasmids p3PS10 and p3PS11 carried the insertion sequence IS*Kpn13*. In contrast, *V. alginolyticus* strain Vb1978 did not show any associated insertion elements. These findings suggest that the variation in mobile genetic elements among the plasmids may be influenced by geographical origin and host species, in contrast to module B, whose composition was conserved irrespective of origin or species.

A comparison of module A of pRAS2 (PV974813) with plasmids from other bacterial species showed conservation of core replication, partitioning and maintenance genes, although variation was observed in accessory elements (Table 7). pRAS2-1682/92 encoded additional genes, including *tnpA* and *sprT*, which were absent only from *p4PS9* (AY6532B). Further differences were observed in the distribution of mobile genetic elements and maintenance-associated genes. For example, pVIM-20710 lacked *bin3* and *trfB*, while *p4PS9* (AY6532B) lacked *trfB*, *higA*, *bin3*, and *prt*, all of which were present in pRAS2. Similarly, Module A of pVb1978 lacked *higA*, *traX*, and *bin3*.

**TABLE 7:** Comparison of module A of pRAS2-1682/92 with other plasmids from different bacteria species.

Gene	PV974813	AY3800B	AY3864B	AY6297B	AY6532B	EIB202	pVIM-20710	Vb1978
Plasmid	(pRAS2)	(p3PS10)	(p3PS11)	(p4PS8)	(p4PS9)	(pEIB202)	(pVIM-20710)	(pVb1978)
*tnpA*	+	+	+	+	-	+	+	+
*sprT*	+	+	+	+	-	+	+	+
*res*	+	+	+	+	+	+	+	+
*korB*	+	+	+	+	+	+	+	+
*soj*	+	+	+	+	+	+	+	+
*trfB*	+	+	+	+	-	+	-	+
*smc*	+	+	+	+	+	+	+	+
*klcA*	+	+	+	+	+	+	+	+
*repA*	+	+	+	+	+	+	+	-
*higA-1*	+	+	+	+	-	+	+	-
*traX*	+	+	+	+	+	+	+	-
*bin-3*	+	+	+	+	-	+	-	-
*prt*	+	+	+	+	-	+	+	+

These observations indicate that although the core replication and maintenance functions of Module A are conserved across these plasmids, individual accessories and stability genes are variably retained.

### 3.7 Characterization of pRAS3 from *Aeromonas salmonicida* donors (1682/92 and 2131/90)

Table 8 lists the pRAS3 plasmids compared in this analysis: those sequenced here from strains 1682/92 and 2131/90 and from the 1682/92-DH5α transconjugant, alongside published *A. salmonicida* pRAS3 sequences from GenBank representing different geographical regions. The genome organization of these plasmids is illustrated in Figure 9. Comparative analysis showed that the pRAS3 plasmids from the donor strains 1682/92 and 2131/90 shared a conserved set of mobilization (MOB) and associated genes, including *mobA*, *mobB*, *mobC*, *mobD*, *mobE* and *ofxX*, as well as *mazF* and *repA*, together with the tetracycline resistance genes interspersed with hypothetical protein-coding genes (Figure 9).

**TABLE 8:** Comparison of pRAS3 from 1682/92 with other plasmids.

Plasmid	Strain	Accession	Species	Location/Year
pRAS3	1682/92	PX927060.1	*Aeromonas salmonicida*	Norway/1992
pRAS3	2131/90	PX927061.1	*Aeromonas salmonicida*	Norway/ 1990
I	OY731307	OY731307.1	*Aeromonas salmonicida*	United Kingdom/2015
pRAS3-3759	SHY18-3759	ON814113.1	*Aeromonas salmonicida*	Canada/2015
pRAS3.1	7067/91, 935/80	AY043298.1	*Aeromonas salmonicida*	Norway/1991 and 1980
pRAS3.3	2009-144K3	KJ909291.1	*Aeromonas salmonicida*	Canada/2009
pRAS3.4	96049	ON814114.1	*Aeromonas salmonicida*	Canada/2015
pRAS3.5	28516	ON814115.1	*Aeromonas salmonicida*	Canada/2015
pRAS3.2	7067/91, 935/80	AY043299.1	*Aeromonas salmonicida*	Norway/1991 and 1980
pRAS3	1682/92-DH5α	This study	*E. coli* transconjugant	Norway

**FIGURE 9:**
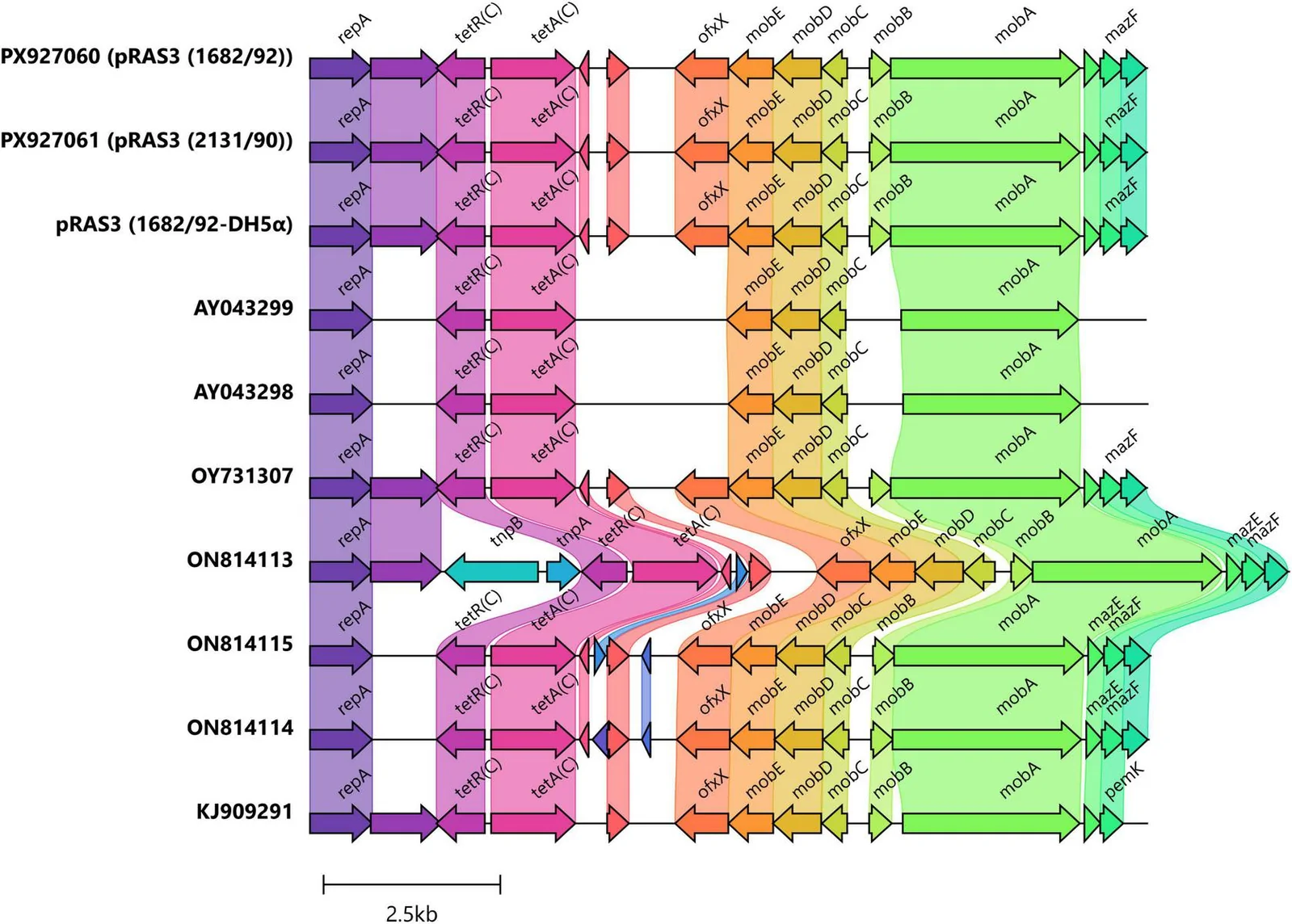
Comparison of the pRAS3 plasmids of *Aeromonas salmonicida* strains PX927060 (1682/92), PX927061 (2131/90), (1682/92-DH5α), AY043299, AY043298, OY731307, ON814113, ON814115, ON814114, KJ909291.

BLAST analysis showed that the pRAS3 sequence in the 1682/92-DH5α transconjugant was nearly identical in gene organization and sequence composition to the OY731307 plasmid from the *A. salmonicida* Hooke isolate (Figure 9). In contrast, pRAS3.4 (ON814114) and pRAS3.5 (ON814115) exhibited a conserved overall gene organization but differed from both donor plasmids (1682/92 and 2131/90) by the presence of three *mazF* genes located between *mobA* and *repA*.

The pRAS3.1 (AY043298) and pRAS3.2 (AY043299) should represent the same plasmids as in our study but weaker annotation in the old study is possibly explaining the differences seen in Figure 9.

Overall, these results indicate that while the core MOB gene organization of pRAS3 plasmids is conserved, substantial variation exists in accessory gene content and arrangement, highlighting the modular nature of this plasmid family.

### 3.8 Whole genome sequencing of the transconjugants after conjugation experiments

Figure 9 and Table 9 show that the pRAS3 of both donor *A. salmonicida* strains 1682/92 and 2131/90 had MOB genes while the recipient *E. coli* DH5α did not have MOB genes prior to conjugation. The transconjugant 1682/92-DH5α derived from mating with the donor strain 1682/92 carrying both pRAS2 and pRAS3, contained all MOB and AMR genes, including *mobA, mobB, mobC, mobD, mobE*, *ofxX*, and *repA* together with the genes for the TetA(C) efflux pump and the TetR(C) repressor, showing a gene organization similar to that of the donor strain 1682/92 (PX927060) (Figure 9 and Table 9). Overall, only the donor strain 1682/92, which encoded both pRAS2 and pRAS3, was able to transfer pRAS3 to the *E. coli* DH5α recipient. In contrast, the donor strain 2131/90, which carried only pRAS3, did not result in transfer to the recipient. These findings indicate that pRAS3 is mobilizable and that its transfer, under the conditions tested, occurred only in the presence of the conjugative plasmid pRAS2 into new DH5α recipient hosts.

**TABLE 9:** Comparison of the MOB genes detected in the donor and transconjugant strains, the *E. coli* DH5α reference assembly, and pRAS3 plasmids from other *A. salmonicida* isolates.

Category	Strain	*repA*	*mobA*	*mobB*	*mobC*	*mobD*	*mobE*	*ofxX*
Donors								
*Aeromonas salmonicida*	1682/92	+	+	+	+	+	+	+
*Aeromonas salmonicida*	2131/90	+	+	+	+	+	+	+
Recipient								
*Escherichia coli*	DH5α	-	-	-	-	-	-	-
Transconjugant								
	1682/92-DH5α	+	+	+	+	+	+	+
Others								
*Aeromonas salmonicida*	AY043298	+	+	–	+	+	+	-
*Aeromonas salmonicida*	AY043299	+	+	–	+	+	+	-
*Aeromonas salmonicida*	KJ909291	+	+	+	+	+	+	+
*Aeromonas salmonicida*	ON814113	+	+	+	+	+	+	+
*Aeromonas salmonicida*	ON814114	+	+	+	+	+	+	+
*Aeromonas salmonicida*	ON814115	+	+	+	+	+	+	+
*Aeromonas salmonicida*	OY731307	+	+	+	+	+	+	+

### 3.9 Historical wet-lab documentation of pRAS2 self-transfer and pRAS2-pRAS3 co-transfer

The whole-genome characterization above was complemented by an extensive series of conjugation trials performed with the same *A. salmonicida* donors between 1993 and 1995, before whole-genome sequencing was available (Table 10). In these experiments, *A. salmonicida* subsp. *salmonicida* strains 1682/92 and 2298/92, both carrying pRAS2 together with pRAS3, were mated with *E. coli* DH5α, and transconjugants were selected on tetracycline or compound sulphonamide and characterized by agarose-gel plasmid profiling and by acquired-resistance testing with antibiotic tablets (Figure 10) and acquired resistance testing; full procedures are described in (L’Abée-Lund and Sørum, 2000). Selection on sulphadiazine, a determinant (*sul2*) carried by pRAS2, recovered transconjugants at high frequency (1.8 × 10^−2^ to 1.3 × 10^−1^ on agar for both donors), and gel profiling of these transconjugants repeatedly showed pRAS2 transferred on its own. Selection on tetracycline since both pRAS2 [*tetA*(31)] and pRAS3 [*tetA*(C)] encode tetracycline resistance resolved two distinct transconjugant populations on the same selection plates in a trial with mating in broth from March 1995: an early-appearing population of larger colonies carrying both pRAS2 and pRAS3, recovered at approximately 1.3 × 10^−7^ to 2.4 × 10^−7^, and a later-appearing population of smaller colonies carrying pRAS2 alone, recovered at approximately 1.6 × 10^−4^ to 2.2 × 10^−4^ (Table 10). Both classes of colonies were confirmed biochemically and by their acquired-resistance phenotype. pRAS2 was thus transferred autonomously at a frequency approximately three orders of magnitude higher than the frequency at which it co-transferred with pRAS3. Agarose gel plasmid profiling demonstrated that pRAS2 was transferred alone in three of four independent conjugation trials while both pRAS2 and pRAS3 were transferred together in one of four conjugation trials. Across all these trials, pRAS3 entered the recipient only when a conjugative helper (pRAS2 or pRAS1) was present in the donor; it was never transferred from a donor in which it resided alone. Taken together with the whole-genome data for the 1682/92-DH5α transconjugant reported above, these results establish that pRAS2 (like pRAS1) is a self-transmissible conjugative plasmid and that pRAS3 is a mobilizable plasmid dependent on a co-resident helper: a helper-carrying donor gives rise to transconjugants that carry the helper alone (pRAS2, at the highest frequency), the helper together with pRAS3, or pRAS3 alone.

**TABLE 10:** Conjugation of pRAS2 with pRAS3 from *Aeromonas salmonicida* to *Escherichia coli* DH5α, performed 1993–1995.

Date	Mating	Selection	Transconjugant colony appearance	Frequency 1682/92	Frequency 2298/92
18 Jun 1993	Agar	Sulphadiazine (200 mg/mL)	–	1.83 × 10^−2^	–
30 Jun 1993	Agar	Sulphadiazine (200 mg/mL)	–	3.9 × 10^−2^	4.35 × 10^−2^
7 Jul 1993	Agar	Sulphadiazine (200 mg/mL)	–	5.2 × 10^−2^	7.0 × 10^−2^
8 Sep 1993	Agar	Sulphadiazine (200 mg/mL)	–	1.3 × 10^−1^	1.3 × 10^−1^
1 Mar 1995	Broth	Tetracycline (25 mg/mL)	Regular colonies	1 × 10^−7^	1.7 × 10^−7^
7 Mar 1995	Broth	Tetracycline (25 mg/mL)	pRAS2 + pRAS3 (early, large colonies)	1.27 × 10^−7^	2.4 × 10^−7^
7 Mar 1995	Broth	Tetracycline (25 mg/mL)	pRAS2 (late, small colonies)	1.58 × 10^−4^	2.2 × 10^−4^

Frequencies of transfer are based on transconjugants recovered as recorded for donors 1682/92 and 2298/92, respectively. Sulphadiazine selects the pRAS2-encoded *sul2* determinant; tetracycline is encoded by both pRAS2 [*tetA*(31)] and pRAS3 [*tetA*(C)], so tetracycline plates recover both pRAS2 only and pRAS2+pRAS3 transconjugants. “–” indicates not recorded.

**FIGURE 10:**
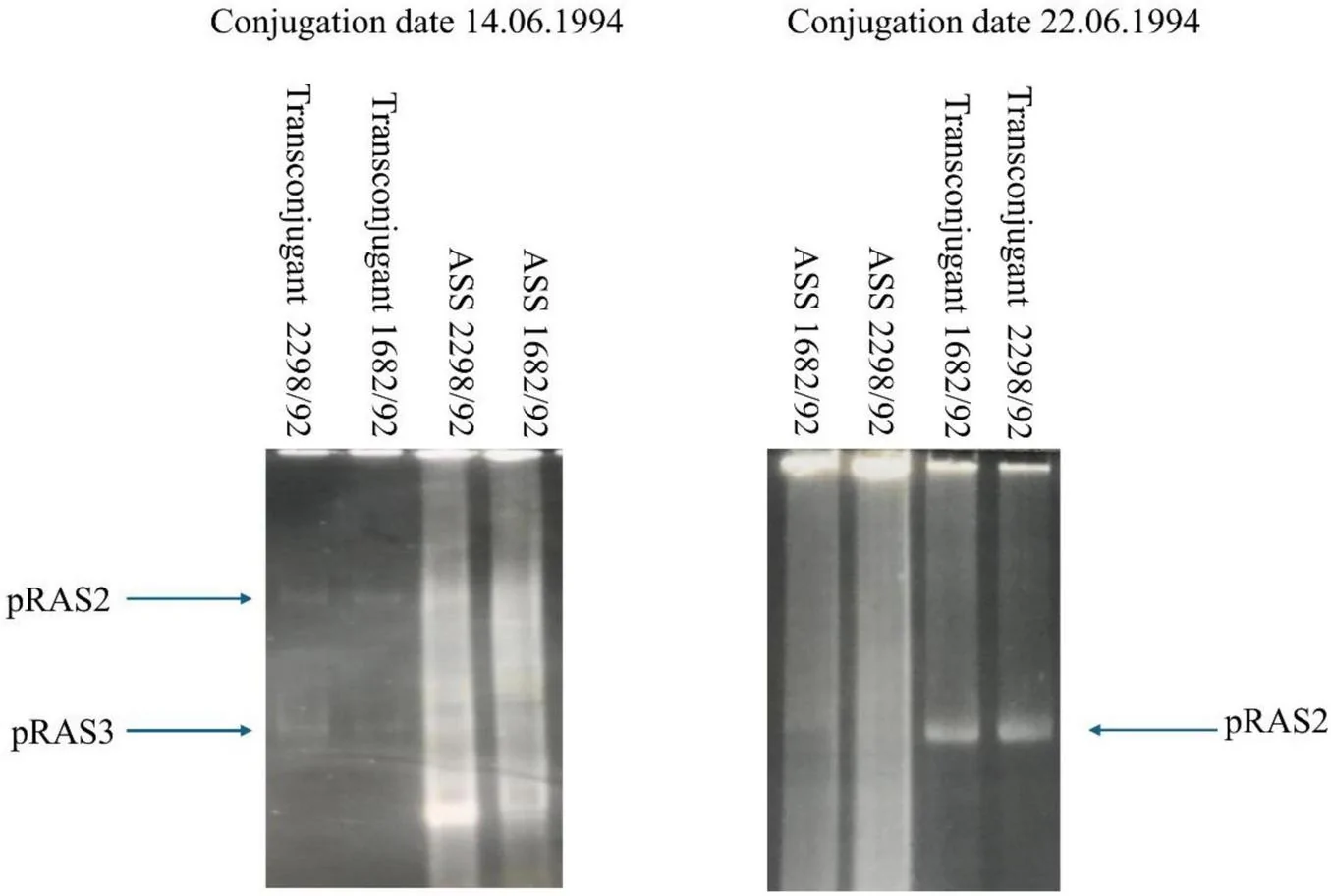
Agarose-gel plasmid profiles documenting transfer of pRAS2 and pRAS3 from *Aeromonas salmonicida* to *Escherichia coli* DH5α. Plasmid DNA was prepared by the Birnboim alkaline-lysis protocol and separated on a 0.7 % agarose vertical gel in TBE buffer at 130 V for 5 h 30 min. Lanes show the *A. salmonicida* donors 1682/92 and 2298/92 (each carrying pRAS2 and pRAS3) and *E. coli* DH5α transconjugants recovered after conjugation (matings of 14 June and 22 June 1994). The positions of pRAS2 and pRAS3 are indicated. Transconjugant profiles show pRAS2 transferred either alone or together with pRAS3, consistent with the frequencies in Table 10.

## 4 Discussion

In this study, we have characterized the pRAS2 and pRAS3 of *A. salmonicida* subspecies *salmonicida* strain 1682/92 and have shown their transfer to *E. coli* DH5α. We have shown that pRAS2 encodes a complete T4SS conjugative apparatus, whereas pRAS3 serves as a mobilizable element dependent on pRAS2 for transfer. Virolle et al. (2020) pointed out that conjugative plasmids carry genes used for vertical transfer from the mother to the daughter cell as well as genes used for plasmid conjugation from the donor to the recipient cell. Collectively, such genes form the backbone structure of the conjugative plasmid.

The composition of genes in module B shows that pRAS2 encodes a canonical T4SS conjugative apparatus seen in other conjugative plasmids (Costa et al., 2021; Ilangovan et al., 2015; Sheedlo et al., 2022). In line with Costa et al. (2021) and Baron (2006) who pointed out that the T4SS nanomachinery in most conjugative plasmids consists of 8–12 conserved proteins, the T4SS of pRAS2 consisted of 10 proteins. Like with other conjugative plasmids (Costa et al., 2021; Ilangovan et al., 2015; Sheedlo et al., 2022), proteins that form the T4SS nanomachinery of pRAS2 can be divided into those that form the extracellular outer membrane (OM) complex, inner membrane (IM) complex, core secretory channel, and cytoplasmic energy complex. Our findings show that pRAS2 has the genes of both virB2/virB5 proteins that form the extracellular OM complex like in other conjugative plasmids in which virB2 forms the bulk of the filamentous pilus structure while virB5 is the minor pilus component whose adhesive properties facilitate contact between donor and recipient cells during conjugation (Backert et al., 2008; Yuan et al., 2005). All virB3/virB6/virB8 proteins that form the IM complex known to provide the structural base of the secretion pore (Mossey et al., 2010) were detected in pRAS2-1682/92. While VirB3 and virB8 are required for synthesis and assembly of the pilus and DNA secretion (Baron, 2006; Jones et al., 1994; Shamaei-Tousi et al., 2004), VirB6 is an endosomal protein that controls the channel and modulates substrate secretion (Mary et al., 2018; Mossey et al., 2010). These results suggest that these proteins play a similar role in the formation and function of the IM complex in pRAS2-1682/92. Of the VirB7/virB9/virB10 proteins that form the core complex secretory channel that spans the periplasm and OM in other conjugative plasmids (Oliveira et al., 2016), only virB9 and virB10 were detected in pRAS2-1682/92. The absence of virB7 was also seen in other plasmid sequences from different bacteria species examined in the present study suggesting that its absence is likely not to hinder the formation of a functional secretory channel in pRAS2-1682/92. While virB9 forms the periplasmic OM component of T4SS (Jakubowski et al., 2005), virB10 forms a stable core complex that connects the OM to the IM thereby bridging the two membranes of the T4SS subassemblies (Christie and Cascales, 2005). Finally, all proteins (virB4/virB11/virD4) that form the cytoplasmic energy complex known to fuel the transport of substrate through the T4SS channel were all detected in pRAS2-1682/92. Cascales and Christie (2004a) pointed out that virB10 is a sensor that detects ATP energy produced by the virD4 and virB11 ATPases. Upon sensing the energy, virB10 undergoes a structural transition that stabilizes the virB7/virB9/virB10 complex, which in turn facilitates easy passage of the DNA substrates through the translocation channel (Cascales and Christie, 2004b). Given that the translocation channel is formed by proteins comprising the VirB3/VirB6/VirB8 inner membrane complex linked to VirB2/VirB5 outer membrane complex, previous studies have shown that VirD4 and VirB11 coordinate ATP-dependent substrate transfer across the cell envelope during conjugation (Cascales and Christie, 2004a). The presence of a complete set of *virB* genes together with *virD4* in pRAS2-1682/92 suggests that this plasmid encodes a canonical type IV secretion system (T4SS) consistent with those described in other conjugative plasmids.

Module B also encodes genes involved in plasmid processing and transfer apart from the T4SS genes. The presence of *topA* that functions in relaxing supercoiled DNA during replication and transcription as well as mediating the cutting and pasting of DNA for transposition (Higgins and Vologodskii, 2015; Sternglanz et al., 1981) indicates that pRAS2-1682/92 can carry out these activities using *topA*. It is likely that the activities of *topA in* pRAS2 are complemented by the presence of *lpd7C*, which belongs to the Large Polyvalent protein-associated Domain (LPD) proteins that are part of the relaxase mobilization system associated with the transfer of plasmid DNA in conjugative transposons and plasmids. The LPD7C relaxases are part of the catalytic domain of the rolling-circle replication (RCR) superfamily that nicks plasmid DNA, forming a covalent linkage with ssDNA, which is then unwound followed by transfer along with the linked relaxase through the T4SS nanomachinery (Tsvetkova et al., 2010; Wallden et al., 2010). Once in the new host, it is re-ligated by the relaxase to form a ssDNA circle (Christie et al., 2014; Llosa et al., 2002). Thus, the presence of *lpd7C* in pRAS2-1682/92 may function in nicking plasmid DNA followed by unwinding and transfer through the T4SS nanomachinery. Moreover, the presence of *cwh* that serves as signal peptide targeted by plasmids that facilitates the translocation or secretion of protein in various bacteria systems (Jomrit et al., 2023; Li et al., 2024) suggests that pRAS2-1682/92 might be using *cwh* for the transfer of plasmid DNA from donor to recipient cells. But the presence of *eexN* that forms the entry exclusion systems aimed at preventing the transfer of plasmid DNA into a host cell that already has the same plasmid indicates pRAS2-1682/92 has a control system that prevents deposition of two or more similar plasmids in one daughter cell. The presence of *traI*, associated with the DNA processing during transfer, together with *traD* that serves as a coupling protein responsible for conjugation, indicates that pRAS2-1682/92 has the transfer functions needed for the self-transfer from one bacterial cell to another.

The composition of genes in pRAS2-1682/92 module A shows it contains genes associated with plasmid replication, maintenance, segregation and protection against host degradation. The presence of *traX* needed for proper F-pilus assembly during conjugation (Maneewannakul et al., 1995) indicates that pRAS2-1682/92 can assemble the F-pilus needed during conjugation to enable efficient DNA transfer between cells. Detection of transposable element *tnpA*, indicates that pRAS2-1682/92 has the conjugation capacity to move and insert its genes into different bacterial cells (Grinsted et al., 1982; Hõrak and Kivisaar, 1998). The presence of *res* which is a member of the resolvase/invertase, and integrase families suggests that pRAS2-1682/92 uses this gene for integration, recombination and maintenance of plasmid DNA after transfer into recipient cells as seen in other conjugative plasmids (Crellin and Rood, 1997). Once integrated into daughter cells, the replication of pRAS2-1682/92 might be facilitated by the help of RepA encoded in its genome given that this gene is useful for starting plasmid DNA replication in daughter cells (Díaz-López et al., 2003; Linder et al., 1985). Encoded by conjugative plasmids, ParA and ParB play key roles in partitioning plasmid copies into daughter cells (Williams and Thomas, 1992). The ParA and ParB family detected in pRAS2 includes *soj*, *korB* and *trfB*. Among these genes, *soj* is critical for partitioning and segregation of plasmids to ensure that daughter cells receive a copy of the plasmid DNA during division (Hester and Lutkenhaus, 2007), while *korB* encodes a transcriptional repressor that coordinates replication, stable inheritance, and transfer of genes that works with the product of *korA* in partitioning and segregation of plasmid copies into daughter cells (Bechhofer et al., 1986). On the other hand, *trfB* serves as a regulator that counteracts the activities of *trfA*. While *trfA* is vital for initiation of plasmid replication, *trfB* limits *trfA* by preventing production of excessive plasmid copy numbers that could kill the host cell. Given that together *soj*, *trfB* and *korB* form a complex maintenance system critical for regulating plasmid copy numbers in daughter cells (Smith et al., 1984; Smith and Thomas, 1984; Theophilus et al., 1985), it is likely that a similar maintenance system exists in pRAS2-1682/92 regulated by presence of these genes. Host cells produce toxic substances that degrade plasmid DNA because it is regarded as foreign when introduced into daughter cells. To subvert this, plasmid DNA must be protected against host cell toxins to ensure survival in daughter cells. The antitoxic genes hosted in pRAS2-1682/92 include *klcA*, and *higA*. The presence of *klcA* could be for protecting pRAS2-1682/92 DNA by functioning as an antidote designed to counteract the toxicity of *klcB* that encodes a toxic factor that kills plasmid DNA (Feng et al., 1994) while the presence of *higA* could be for neutralizing the toxic effect of *higB* ribonuclease not to degrade the plasmid DNA of pRAS2-1682/92 as seen in other plasmids (Schureck et al., 2019; Tian et al., 1996).

Module C primarily encoded antimicrobial resistance (AMR) genes and was the most diverse of the three pRAS2-1682/92 modules when compared with related plasmids. Among the plasmids analyzed, pRAS2-1682/92 along with pEIB202, p3PS10, and p3PS11, carried *tetA*(31) together with the associated regulatory gene *tetR*(31). In contrast, these tetracycline resistance genes were absent in pVb1978 and other plasmids lacking this module. In addition to these shared determinants, each plasmid encoded distinct AMR genes. The Norwegian *A. salmonicida* strain 1682/92 harbored *sul2*, *aph(6)-Id* and *neo*, whereas the Chilean *P. salmonis* plasmids p3PS10 and p3PS11 contained both *sul2*, *ant1*, and *tetA*(31) and *tetR*(31), and p4PS8 contained *cmlA* and *ant1*. In contrast, pEIB202 of the Chinese *E. piscicida* strain encoded *tetA*(31), *tetR*(31), *aph(6)-Id*, *sul2*, *cat3*, and *mobA*, while pVb1978 of *V. alginolyticus* encoded *aacA4*, *ant1*, *sul2*, *cat*, and an *emrE* multidrug transporter but lacked tetracycline resistance genes. These observations indicate that while certain resistance determinants such as *sul2* and tetracycline resistance genes are shared among a subset of plasmids, module C exhibits substantial variation in accessory AMR genes across different bacterial species. This variability is further reflected in the distribution of mobile genetic elements. For example, the Chilean *P. salmonis* plasmids carried the insertion sequence IS*Kpn13* and pEIB202 carried an IS*Prst3* transposase, whereas pVb1978 lacked associated insertion elements. Given that insertion sequences and transposases facilitate the acquisition and rearrangement of resistance genes, these findings suggest that the diversity of AMR genes in module C may be shaped by the activity of distinct mobile genetic elements in different bacterial hosts. In contrast, *topA*, identified in module B across most of the plasmids examined, appears to represent a conserved backbone component associated with replication and maintenance functions.

Finally, in this study we showed that only *A. salmonicida* strain 1682/92, which harbored both pRAS2-1682/92 and pRAS3-1682/92, was able to transfer pRAS3 to the *E. coli* DH5α recipient, resulting in formation of the transconjugant 1682/92-DH5α. The success of transfer was confirmed by the conserved composition and organization of MOB and associated genes in pRAS3 in both the donor strain and the transconjugant. In contrast, no transconjugant colonies were obtained from matings involving strain 2131/90, which carried pRAS3 but lacked pRAS2, indicating that pRAS3 is a mobilizable plasmid whose transfer requires a co-resident conjugative plasmid such as pRAS2.

These findings demonstrate that pRAS2 functions as a helper plasmid, providing the conjugative machinery necessary for pRAS3 transfer. Mobilizable plasmids typically encode only part of the transfer machinery, such as an origin of transfer and relaxase-associated functions and therefore depend on conjugative elements supplied *in trans* for transfer (von Wintersdorff et al., 2016). Plasmid-plasmid cooperation is well documented in several bacterial systems, including Enterobacteriaceae and Vibrionaceae (Lang et al., 2012; Smillie et al., 2010). However, such helper-mediated mobilization has not previously been clearly described for the pRAS plasmid family in *A. salmonicida*. The observation that pRAS3 was mobilized without detectable co-transfer of pRAS2 is therefore noteworthy. Similar phenomena have been reported in *Bacillus subtilis*, where the integrative and conjugative element ICEBs1 can mobilize plasmids lacking dedicated mobilization functions without co-transfer of the conjugative element (Lee et al., 2012; Miyano et al., 2018). Together, these findings suggest that pRAS2 provides transfer functions *in trans*, enabling pRAS3 mobilization while remaining undetected in recipient cells under the conditions tested.

Earlier work (L’Abée-Lund and Sørum, 2000; L’Abée-Lund and Sørum, 2002) reported that pRAS2 was transferred together with pRAS3 into the same *E. coli* DH5α background used here, whereas the transconjugant characterized by whole-genome sequencing in the present study carried pRAS3 without pRAS2. This apparent difference is resolved by the conjugation record summarized in section 3.9 and Table 10. Matings of a pRAS2 + pRAS3 donor with DH5α do not give a single transfer outcome but a mixture: transconjugants carrying pRAS2 alone (recovered at the highest frequency, ∼10^−4^), transconjugants carrying both pRAS2 and pRAS3 (∼10^−7^), and transconjugants carrying pRAS3 alone, in which the helper plasmid remained in the donor or was not maintained in the recipient.

Equivalent behavior was documented for the related conjugative plasmid pRAS1: *A salmonicida* donors carrying pRAS1 together with pRAS3 (strains 151.1/92, 151.2/93, 151.3/92, and the atypical isolate 935/80) transferred pRAS1 alone, pRAS1 with pRAS3, or neither, in the same variable manner (L’Abée-Lund and Sørum, 2000).

The results in this and previous studies indicate that the efficiency of mobilization of pRAS3 by a co-residing conjugable R plasmid as pRAS2 and pRAS1 varies from no transfer to a relatively high frequency. Because tetracycline selection recovers all three transconjugant types and only a single transconjugant colony was taken forward for sequencing here, the recovery of a pRAS3 only transconjugant is consistent with the earlier reports rather than in conflict with them. Several non-exclusive factors are expected to bias which class is sequenced, including approximately three orders of magnitude difference in frequency between autonomous pRAS2 transfer and pRAS2-pRAS3 co-transfer. The direction of antibiotic selection, and the reduced replication or segregational stability expected for a plasmid of a psychrotrophic host propagated in *E. coli* at 37 °C; entry exclusion mediated by *eexN* is not expected to contribute, because the plasmid-free recipient carried no related replicon and *eexN* restricts redundant donor to donor transfer rather than entry into a native recipient. Taken together, the whole-genome data and the conjugation trials in section 3.9 establish that pRAS2 is a self-transmissible conjugative plasmid and that pRAS3 is a mobilizable plasmid whose transfer requires a co-resident helper such as pRAS2 or pRAS1.

The remaining open question concerns the persistence rather than the occurrence of transfer. Serial passage of the transconjugants over successive generations is needed to establish the stability and loss rate of pRAS3 in the *E. coli* background, particularly given the reduced replication and segregational stability expected for a plasmid of a psychrotrophic host propagated at 37 °C.

The identification of pRAS2-like plasmids across geographically distant aquaculture systems highlights their wide distribution and significance. Comparative analysis showed that pRAS2 shares a conserved modular backbone with these plasmids. The closest relative, pBY3 from *A. salmonicida* subsp. *masoucida* in South Korea, shared 99.99% nucleotide identity over 82% query coverage, and the plasmids from Chile and China also showed high nucleotide identity. Conservation was therefore concentrated in the replication, maintenance and T4SS modules, with variation primary in the antimicrobial resistance region. Similarly, pRAS1 related plasmids have been reported in *Edwardsiella piscicida*, further supporting the distribution of related resistance-associated plasmids across economically important aquaculture pathogens (Data not shown).

The occurrence of pRAS2-like plasmids in diverse aquatic bacterial species indicates that a highly conserved conjugative backbone, with near-identical nucleotide sequences across isolates, is circulating in aquaculture-associated environments. Together with the helper-dependent mobilization of pRAS3 observed in this study, these findings support a role for plasmid-plasmid cooperation in the dissemination of AMR genes in aquatic bacterial communities. The presence of such conserved plasmids in *A. salmonicida* in Norway, and in *P. salmonis* in Chile may reflect dissemination linked to aquaculture practices, including the movement of fish stocks. However, the identification of nearly identical plasmid backbone in phylogenetically distinct species such as *Edwardsiella piscicida* and *Vibrio alginolyticus* suggests that these plasmids may also circulate through broader environmental reservoirs, potentially facilitated by large-scale aquatic connectivity such as ocean currents, rather than solely by direct anthropogenic transfer.

Comparative analysis indicates that the conserved modular backbone of pRAS2 is shared, in whole or in part, by plasmids from Norway, South Korea, Chile and China, with variation primarily restricted to the antimicrobial resistance region. Similar observations have been reported for related plasmids, including the IncU plasmid pRAS1, which shares an identical backbone with the plasmid RA3 isolated decades earlier from aquaculture environments in Japan (Aoki et al., 1971; Sørum et al., 2003). In addition, pRAS4-like plasmids identified in environmental samples lacking AMR determinants, compared with AMR-associated variants in Norwegian isolates, suggest acquisition of resistance genes over time (Dahal et al., 2026). Together, these findings indicate that pRAS plasmids are widely distributed across diverse bacterial hosts and aquaculture environments and may serve as important vehicles for the dissemination of antimicrobial resistance in globally significant fish production systems. So far it may be suggested that identical conjugative plasmids exist in marine and freshwater systems all around the globe as part of the aquatic microbiota. Use of antimicrobial drugs in aquaculture may be an important driver of these conjugative plasmids when they attract and incorporate drug resistance suited to protect the fish pathogens against the antibiotic drugs used in the local fish farming system to control infectious bacterial diseases as furunculosis in salmonids. This speculation fits with an increasing awareness that aquatic microbiota may be a crucial platform for recruiting AMR that ends into the human clinical facilities and their patients.

## 5 Conclusion

In this study we have shown that pRAS2 of *A. salmonicida* strain 1682/92 encodes a canonical T4SS nanomachinery capable of transferring plasmid DNA from donors to recipient bacteria. We have also shown that its genome is divided into three modules encoding, respectively, plasmid replication and maintenance functions (Module A), the T4SS conjugation machinery (Module B), and the antimicrobial resistance genes with their associated mobile genetic elements (Module C). Finally, we demonstrated that pRAS2 is a self-transmissible conjugative plasmid that also serves as a helper for the mobilization of the non-conjugative pRAS3. Conjugation trials show that pRAS2 transfers autonomously at high frequency and co-mobilizes pRAS3 less efficiently, so that a pRAS2 + pRAS3 donor yields transconjugants carrying pRAS2 alone, pRAS2 with pRAS3, or pRAS3 alone. Thus, pRAS2 represents a potential driver for the spread of AMR both through its conjugative transfer system and by facilitating the mobilization of non-conjugative plasmids like pRAS3.

## Data Availability

The datasets presented in this study can be found in online repositories. The names of the repository/repositories and accession number(s) can be found at: https://www.ncbi.nlm.nih.gov/, PV974813; https://www.ncbi.nlm.nih.gov/, PX927060.1; https://www.ncbi.nlm.nih.gov/, PX927061.1.
